# Activation of Secondary Metabolism in Citrus Plants Is Associated to Sensitivity to Combined Drought and High Temperatures

**DOI:** 10.3389/fpls.2016.01954

**Published:** 2017-01-09

**Authors:** Sara I. Zandalinas, Carlos Sales, Joaquim Beltrán, Aurelio Gómez-Cadenas, Vicent Arbona

**Affiliations:** ^1^Departament de Ciències Agràries i del Medi Natural, Ecofisiologia i Biotecnologia, Universitat Jaume ICastelló de la Plana, Spain; ^2^Departament de de Química Física i Analítica, Research Institute for Pesticides and Water, Universitat Jaume ICastelló de la Plana, Spain

**Keywords:** Carrizo citrange, Cleopatra mandarin, citrus, abiotic stress combination, metabolome

## Abstract

Drought and heat stresses are two of the most frequent environmental factors that take place simultaneously in the field constraining global crop productivity. Metabolism reconfiguration is often behind the adaptation of plants to adverse environmental conditions. Carrizo citrange and Cleopatra mandarin, two citrus genotypes with contrasting ability to tolerate combined heat and drought conditions, showed different metabolite patterns. Increased levels of phenylpropanoid metabolites were observed in Cleopatra in response to stress, including scopolin, a metabolite involved in defense mechanisms. Tolerant Carrizo accumulated sinapic acid and sinapoyl aldehyde, direct precursors of lignins. Finally, Cleopatra showed an accumulation of flavonols and glycosylated and polymethoxylated flavones such as tangeritin. The activation of flavonoid biosynthesis in Cleopatra could be aimed to mitigate the higher oxidative damage observed in this genotype. In general, limonoids were more severely altered in Cleopatra than in Carrizo in response to stress imposition. To conclude, all metabolite changes observed in Cleopatra suggest the activation of energy metabolism along with metabolic pathways leading to the accumulation of photoprotective and antioxidant secondary metabolites, oriented to mitigate the damaging effects of stress. Conversely, the higher ability of Carrizo to retain a high photosynthetic activity and to cope with oxidative stress allowed the maintenance of the metabolic activity and prevented the accumulation of antioxidant metabolites.

## Introduction

Plant mechanisms for acclimation and adaptation to challenging environmental conditions are based on the activation of specific physiological and molecular responses. These responses, in turn, lead to changes in plant metabolism to minimize stress-induced damage. Studies focusing on a single abiotic factor do not represent the particular response of plants to a combination of different stresses, likely affecting to crops growing in the field (Mittler and Blumwald, 2010; Suzuki et al., 2014; De Boeck et al., 2015; Hu et al., 2015; Liu et al., 2015; Zhang et al., 2015). Specifically, the combination of drought and heat stress is considered one of the most recurrent condition that takes place in natural environments, affecting plant growth and productivity (Savin and Nicolas, 1996; Jiang and Huang, 2001; Mittler, 2006; Craufurd et al., 2008; De Boeck et al., 2015; Zandalinas et al., 2016b). In order to face combinations of drought and heat stress, plants specifically alter gene expression in a very different way that changes occurring in plants grown under defined stress conditions (drought or heat applied individually) (Rizhsky et al., 2002, 2004; Mittler, 2006). These alterations in gene expression lead to a specific regulation of the metabolome depending on the particular stress and the plant species (Rizhsky et al., 2004; Sun et al., 2016).

In our previous work (Zandalinas et al., 2016b), we studied the particular physiological response of two citrus genotypes, Carrizo citrange and Cleopatra mandarin, to the combined action of water deficit and high temperatures. In that work, stress combination imposed a more damaging situation for citrus plants, as previously reported in other plant species such as *Triticum aestivum* (Wang et al., 2010), *Nicotiana tabacum* (Rizhsky et al., 2002), *Populus yunnanensis* (Li et al., 2014), and *Arabidopsis thaliana* (Rizhsky et al., 2004; Zandalinas et al., 2016a). However, Carrizo was found to be more tolerant to high temperatures applied alone or in combination with water deprivation than Cleopatra. Chlorophyll fluorescence data evidenced the damaging influence of heat stress on the ability of Cleopatra plants to photosynthesize. In this genotype, PSII performance (F_v_/F_m_) and photosynthetic electron flow (Φ_PSII_) significantly decreased in response to heat and combined conditions of drought and heat, whereas PSII values in Carrizo plants were affected only by the stress combination. Furthermore, higher transpiration and photosynthetic rates along with reduced oxidative damage were also identified as additional traits behind this increased tolerance (Table S1). Nonetheless, metabolic changes of these citrus plants could also contribute to this contrasting ability to tolerate different abiotic stresses applied individually or in combination.

Plant metabolome comprises a huge diversity of low molecular weight compounds, including carbohydrates, acids, amino acids, phenolics, polyols, polyamines, lipids, and others, with many different biological functions. This diversity of metabolites is usually divided into primary metabolism, including different polar metabolites such as carbohydrates, as direct products of photosynthesis and substrates of the energy metabolism; tricarboxylic acid cycle (TCA) intermediates, such as citric acid or oxalacetic acid (OAA); amino acids involved in protein synthesis, or other cell processes such as osmotic readjustment (e.g., proline); etc. Different studies comparing polar compound accumulation in plants subjected to abiotic stresses such as drought, salinity, excess light or low and high temperatures, have identified metabolites involved in plant acclimation to these abiotic stresses (Kaplan et al., 2004; Cramer et al., 2007; Maruyama et al., 2009; Caldana et al., 2011). For instance, TCA cycle, gluconeogenesis and photorespiration are activated under osmotic stress, leading to increased glucose, malate, and proline levels to adjust osmotically and to cope with ROS production and photoinhibition (Cramer et al., 2007).

Secondary metabolites such as phenylpropanoids and their derivatives are an important group of compounds essential for plant acclimation and survival to varying environmental conditions, including coumarins, lignin building blocks, flavonoids, anthocyanins, and tannins (Fraser and Chapple, 2011). They are usually semi-polar compounds and have a variety of physiological roles including ROS scavenging, enzyme activation, photoprotection and signal regulation (Dixon and Paiva, 1995; Zhao et al., 2005; Naoumkina et al., 2010; Arbona et al., 2013). In addition, limonoids are naturally-occurring secondary metabolites derived from isoprenoids found in plant species of the Rutaceae and Meliaceae families whose antioxidant activity has been previously demonstrated (Yu et al., 2005; Perez et al., 2009).

This wide variety of compounds makes difficult the selection of a single exhaustive analytical technology to attain metabolome analysis. The most popular analytical platforms are based on mass spectrometry (MS) coupled to a separation technique such as ultra-performance liquid chromatography (UPLC), gas chromatography (GC), or capillary electrophoresis. Using GC-MS, many compounds with different chemical composition including organic acids, sugars, amino acids, sugar alcohols, aromatic amines, or TCA cycle intermediates can be profiled and identified by matching spectra with available spectral libraries (Shulaev et al., 2008). In addition, another useful metabolite profiling technique to analyze semi-polar and non-polar metabolites is UPLC coupled to hybrid quadrupole time-of-flight mass spectrometers (QTOF) (Von Roepenack-lahaye et al., 2004).

Therefore, the aim of this study was to analyze the impact of drought and heat, two major abiotic stresses, acting isolated from each other or in combination, on primary and secondary metabolisms of Carrizo citrange and C. mandarin and to correlate the differential metabolite accumulation with the contrasting tolerance to adverse situations.

## Methods

### Plant material and growth conditions

True-to-type Carrizo citrange (*Poncirus trifoliata* L. Raf. x *Citrus sinensis* L. Osb.) and C. mandarin (*Citrus reshni* Hort. Ex Tan.) plants were purchased from an authorized commercial nursery (Beniplant S.L., Penyíscola, Spain). One-year-old seedlings of the two citrus genotypes were individually placed in plastic pots filled with perlite and watered three times a week with 0.5 L of a half-strength Hoagland solution. Plants were grown in the greenhouse under natural photoperiod and day and night temperature averaging 25.0 ± 3.0°C and 18.0 ± 3.0°C, respectively, for 1 month to allow acclimation. Subsequently, plants were maintained in growth chambers under a 16-h photoperiod (light fluency of 300 μmol m^−2^ s^−1^ PPF), 25 °C and a relative ambient moisture of 80% for 2 weeks before the beginning of the experiments. Temperature and relative moisture were recorded regularly with a portable USB datalogger (OM-EL-WIN-USB, Omega, New Jersey, USA).

### Stress treatments and experimental designs

A 24-h experiment was performed in which severe drought, imposed by transplanting plants to dry perlite, was applied alone or in combination with high temperatures (40°C). Prior to imposition of drought, heat stress was applied for 7 days to a group of well-watered Carrizo and Cleopatra plants whereas another group was maintained at 25°C. Thereby, we established four experimental groups for each genotype: Well-watered plants grown at 25°C (CT) and 40°C (HS) and plants subjected to drought at 25 °C (WS) and at 40°C (WS+HS). Fully expanded leaves with an intermediate position in the canopy were harvested 24 h after the imposition of water stress by performing a gentle cut at the petiole with a scalpel. Harvesting was performed during day time and illuminated leaves were immediately submerged in liquid N_2_ to deter all metabolic activity. Frozen leaves were crushed and ground to fine powder and stored at −80°C for subsequent metabolomic analyses.

### GC-MS extraction and derivatization

For primary metabolite analysis by GC-MS, 50 mg of fresh leaf tissue was extracted in 300 μL of pure methanol (LC-MS grade, Panreac, Barcelona, Spain) spiked to 0.2 mg ml^−1^ with ribitol as internal standard (IS), following Roessner et al. (2001). Extractions were performed by ultra-sonication for 10 min at room temperature. After centrifugation, supernatants were recovered and mixed with 200 μL of chloroform and 400 μL of water and centrifuged at 10000 rpm and 4°C for 10 min to allow phase separation. The upper water layer was recovered and evaporated to dryness using a SpeedVac (Jouan, Saint HerblainCedex, France). Dry residues were re-dissolved with 50 μL of 20 mg mL^−1^ methoxamine in pyridine followed by incubation at 30°C in a water bath for 90 min. Later, 70 μL of methylsilyltrifluoroacetamide (Macherey-Nagel, Germany) were added and subsequently incubated at 37°C for 30 min. Finally, the solution was mixed with 10 μL of a commercial mixture of fatty acid methyl esters (C8-C24 FAME mix, Sigma-Aldrich, Madrid, Spain) as retention index (RI) markers.

### Instrumental conditions for GC-MS analysis

Derivatized extracts were independently injected onto a GC-MS system composed of a gas chromatograph equipped with an autosampler and a quadrupole mass analyzer (GCMS-QP2010 Ultra, Shimadzu Corporation, Japan) equipped with an electron impact ion source. The GC separation was performed using a fused silica BPX5 capillary column with a length of 30 m × 0.25 mm ID and a film thickness of 0.25 μm (SGE Analytical Science, Victoria, Australia). The oven program was set as follows: 80°C (2 min); 10°C min^−1^ to 325°C (3.5 min) for a total runtime of 30 min. Injections of 1 μL of sample extracts were performed in split mode (1:200) at a temperature of 230°C. Helium (99.999 %; Praxair, Valencia, Spain) was used as the carrier gas at a constant flow rate of 2 mL min^−1^. The interface and source temperatures were set to 325°C and 230°C, respectively. Scan rate was set at 5 scan s^−1^ within 50 to 700 amu mass range. After acquisition, chromatogram files were converted to NetCDF for further processing.

### GC-MS data processing

Mass chromatographic features were extracted with xcms (Smith et al., 2006) and subsequently processed with TargetSearch software (Cuadros-Inostroza et al., 2009) using Golm Metabolite Database (available from http://www.mpimp-golm.mpg.de/). Briefly, this software calculates RI for all compounds in chromatograms based on tabulated RI values for the constituents of the FAME mix and then matches RI and mass fragments of compounds in samples with those found in databases. Identified metabolites were cross-referenced in chromatograms using GCMS-solution software (Shimadzu Corp.). Peak areas of identified metabolites were normalized to the IS (ribitol) area before statistical analyses.

### LC-MS extraction

Extraction was performed essentially as previously described in Zandalinas et al. (2012) with slight modifications. Briefly, 500 μL of 70% methanol supplemented with biochanin A at 1 mg L^−1^ (IS) was added to 0.1 g of frozen leaf powder. After 10 min of sonication, extracts were heated at 80°C in a water bath for 15 min and allowed to cool down at room temperature. Then, samples were centrifuged at 10000 g for 10 min at 4°C and supernatants filtered through 0.22 μm PTFE syringe filters (Whatman International Inc., Kent, United Kingdom), prior to UPLC/ESI-QTOF-MS analysis.

### LC-MS instrumental conditions

Chromatographic separations were performed on an Acquity SDS system (Waters Corp. Ltd., Milford, MA) interfaced to a QTOF Premier from Micromass Ltd. through an ESI source. A reversed-phase column was used as follows: 100 mm × 2.1 mm i.d., 1.8 μm, ProntoSil, Bischoff, Leonberg, Germany). Sample aliquots (10 μL) were injected onto the UPLC system using the partial loop-filling option. Separations were carried out using a flow rate of 300 μL min^−1^ for 30 min as follows: 0−2 min, isocratic 95% A [water:formic acid, 99.9:0.1 (v/v)] and 5% B [acetonitrile: Formic acid, 99.9:0.1 (v/v)]; 2−17 min, gradient 5−95% B; 17−20 min, return to initial conditions; 20−25 min, re-equilibration period. During analyses, the column temperature was maintained at 40°C and samples were maintained at 10°C to slow down degradation. Samples were analyzed in both negative and positive ionization modes. Two functions were set in the instrument: In function 1, data were acquired in profile mode from 50 to 1000 Da using a scan time of 0.2 s and a collision energy of 2 eV; in function 2, the scan range was the same, but a collision ramp between 4 and 65 eV was set. During all measurements, the electrospray capillary voltage was set to 4 kV and the cone voltage was set to 25 V. The source temperature was maintained at 120°C and the desolvation gas temperature was set at 350°C. Argon was used as the collision gas and nitrogen was used as the nebulizer as well as desolvation gas set at 60 and 800 L h^−1^, respectively. Exact mass measurements were provided by monitoring the reference compound lockmass leucine-enkephalin.

### LC-MS data processing

Data were processed using Masslynx v.4.1. Raw data files were converted to netCDF format using the application databridge from Masslynx and processed using the xcms package as previously described (Smith et al., 2006; Zandalinas et al., 2012). Chromatographic peak detection was performed using the matchedFilter algorithm, applying the following parameter settings: snr = 3, fwhm = 15 s, step = 0.01 D, mzdiff = 0.1 D, and profmethod = bin. Retention time correction was achieved in three iterations applying the parameter settings minfrac = 1, bw = 30 s, mzwid = 0.05 D, span = 1, and missing = extra = 1 for the first iteration; minfrac = 1, bw = 10 s, mzwid = 0.05 D, span = 0.6, and missing = extra = 0 for the second iteration; and minfrac = 1, bw = 5 s, mzwid = 0.05 D, span = 0.5, and missing = extra = 0 for the third iteration. After final peak grouping (minfrac = 1, bw = 5 s) and filling in of missing features using the fillPeaks routine of the xcms package, a data matrix consisting on feature × sample was obtained.

### Statistical analyses

Significantly altered metabolites were extracted from datasets following ANOVA analysis at *p* ≤ 0.05 using sample treatment as factor. To investigate metabolite profiles over samples, genotypes and treatments, peak areas of significantly altered metabolites were represented as a heatmap to facilitate visualization. Both heatmaps and hierarchical cluster analysis (HCA) on heatmaps were performed using Dchip software (http://www.hsph.harvard.edu/cli/complab/dchip/). PLS-DA was performed with Simca-P+ 11.0 (Umetrics AB, Umeá, Sweden). In addition, metabolite levels were subjected to analysis of variance using a two-way ANOVA considering the two citrus genotypes and the four stress treatments followed by Tukey *post-hoc* test (*p* < 0.05) when a significant difference was detected (Tables S2, S3).

## Results

### Analysis of polar metabolites

**Figure 1:**
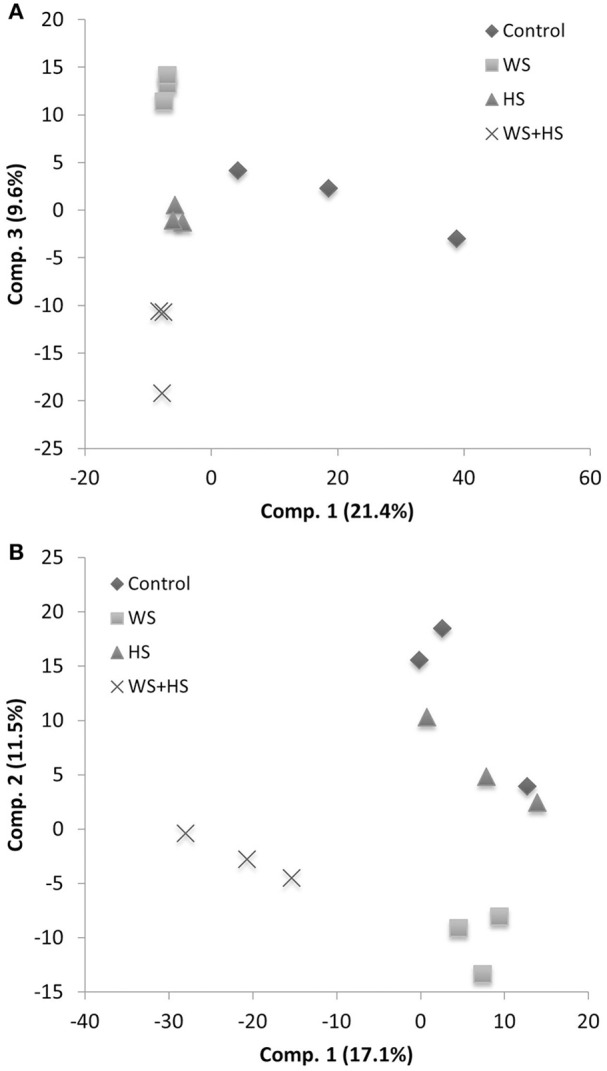
**Partial Least Squares-Discriminant Analysis (PLS-DA) of polar metabolite profiles in Carrizo (A)** and Cleopatra **(B)**.

**Figure 2:**
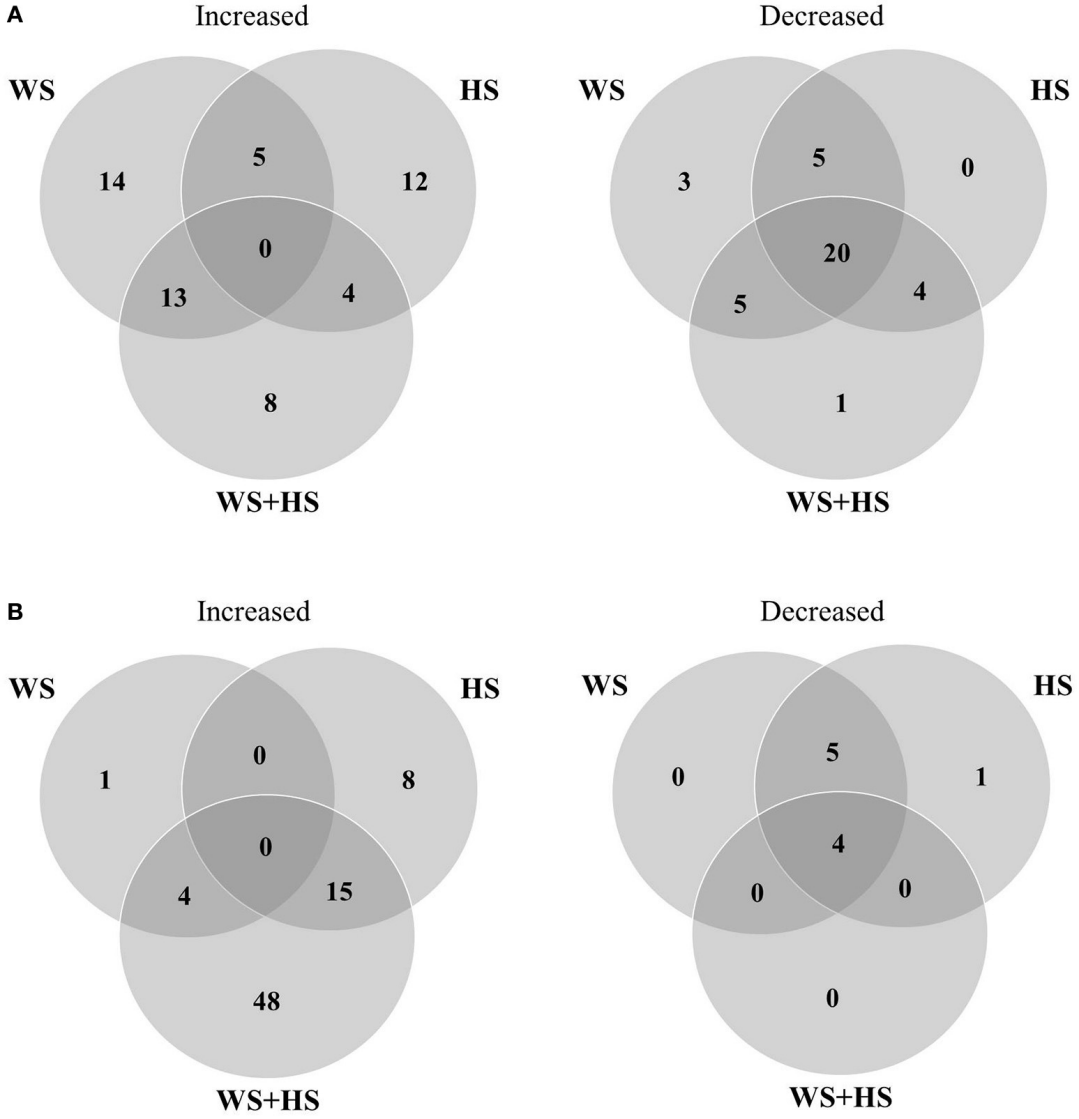
**Venn diagrams depicting overlapping polar metabolites significantly altered during WS, HS and WS+HS in Carrizo (A)** and Cleopatra **(B)**.

Non-targeted GC-MS analysis was performed to determine different patterns of polar metabolite accumulation in leaves of citrus plants subjected to WS, HS, and WS+HS. Results revealed different metabolite profiles in both citrus genotypes under individual and combined stress conditions (Figures 1, 2). In Carrizo, stress was the main source of variability (21.4%) allowing the differentiation of a “control” cluster of samples from the rest of treated samples. Within this “stressed” cluster, all three treatments (WS, HS, and WS+HS) could be easily differentiated (Figure 1A). Conversely, the main source of variability (17.1%) of polar metabolites in Cleopatra was the imposition of combined stress conditions (WS+HS) with a minor contribution of WS (11.5%). Interestingly, control samples could not be clearly differentiated from HS samples (Figure 1B). Analysis of variance revealed a total of 94 polar metabolites differentially altered (56 accumulated and 38 decreased, out of 1143 detected) in Carrizo plants of which 14 were accumulated only during WS (Figure 2A). Only 8 compounds were found specifically altered in Carrizo during WS+HS and heat stress alone induced the accumulation of 12 compounds (Figure 2A). Stress imposition significantly altered a total of 20 metabolites whereas the impact of individual stresses depressing metabolite levels was more restricted (Figure 2A).

In Cleopatra, stress imposition significantly altered a total of 78 polar metabolites out of 1113 detected in GC-MS analyses, of which 68 were accumulated and 10 reduced (Figure 2B). Stress combination induced the accumulation of 48 metabolites including the glycolytic end-product pyruvate as well as the TCA intermediate succinate, whereas WS applied alone induced the accumulation of only 1 polar metabolite in Cleopatra. Both HS and WS+HS stresses caused the increase in levels of 15 metabolites (Figure 2B). As mentioned above, stress imposition did not have such a strong impact on depressing polar metabolite levels in Cleopatra compared to Carrizo (levels of 4 metabolites decreased upon stress imposition, 5 compounds shared by HS and WS and only one was significantly altered by HS applied alone) (Figure 2B).

### Accumulation of polar metabolites: glycolysis-TCA cycle

To investigate differences between genotypes in the accumulation of different polar compounds, metabolite levels were represented within their respective pathways.

**Figure 3:**
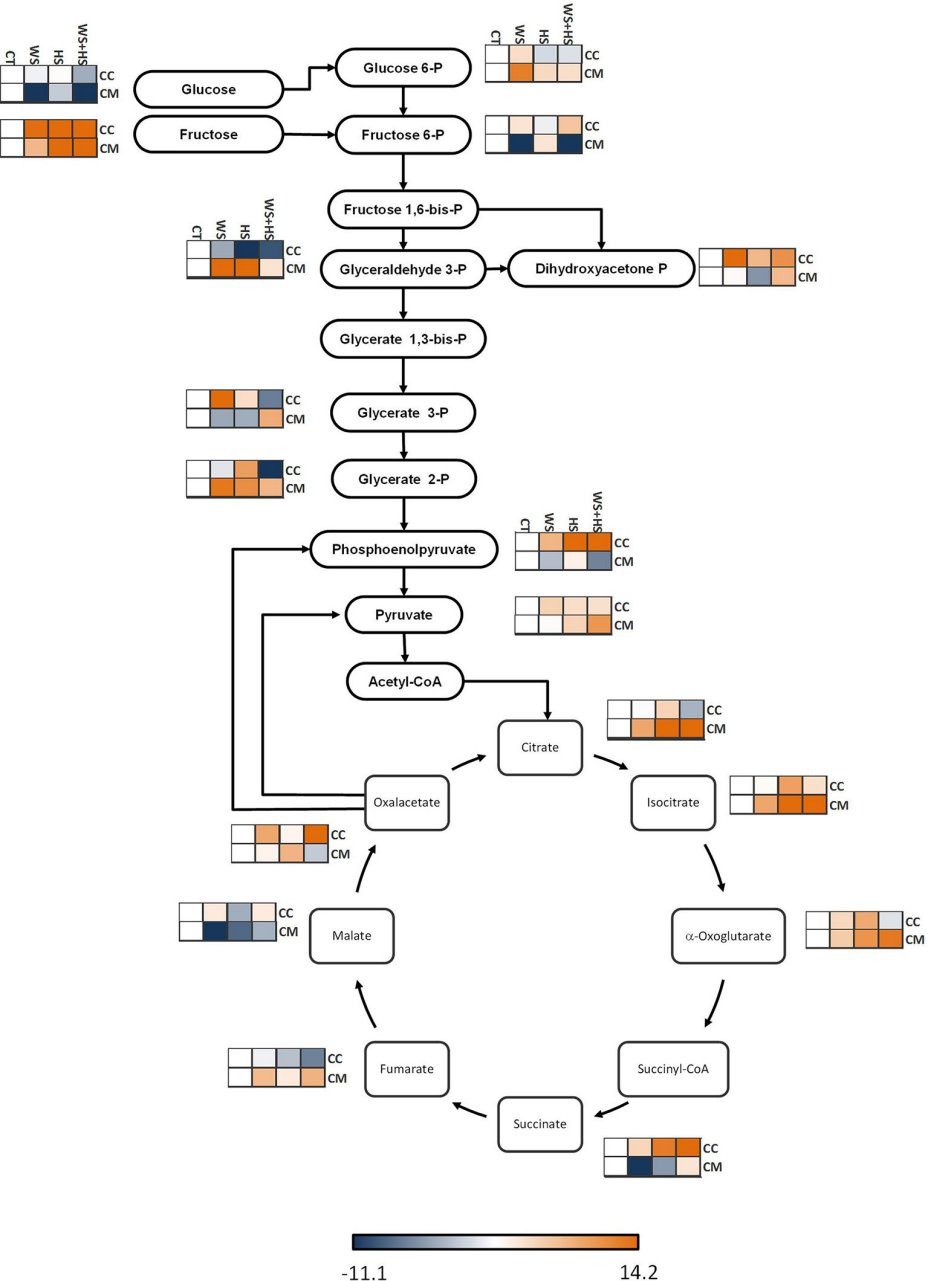
**Levels of metabolites involved in glycolysis and TCA cycle**. Relative levels [expressed as log_2_ (stress/control)] are given besides each detected metabolite as a heatmap: CC, Carrizo and CM, Cleopatra, CT, control; WS, water stress; HS, heat stress; WS+HS, stress combination. Statistical analyses for each metabolite are given in Table S2.

Carrizo and Cleopatra displayed different accumulation of metabolites related to glycolysis and TCA cycle depending on the stress type. As a general trend, stress imposition induced fructose accumulation in both citrus genotypes, especially in response to HS and WS+HS (Figure 3). In addition, WS induced a significant glucose-6-P accumulation in Cleopatra. On the contrary, in this genotype, fructose-6-P levels were depleted in Cleopatra plants subjected to WS or stress combination, in contrast to the response observed in Carrizo (Figure 3). Both genotypes also showed differences at the level of fructose 1,6-bis-P splitting catalyzed by aldolase: Cleopatra preferentially accumulated glyceraldehyde 3-P in response to WS and HS whereas Carrizo showed higher levels of dihydoxyacetone-P in response to all stress treatments but especially WS. Glycerate 3-P levels increased specifically in response to WS in Carrizo and, to a lesser extent in response to HS, whereas WS+HS reduced its levels. In Cleopatra, the sensitive genotype, individual stress imposition reduced glycerate 3-P levels but stress combination significantly increased them. On the other hand, glycerate 2-P showed increased levels in response to HS in Carrizo and in response to WS and HS in Cleopatra. In addition, stress combination reduced its levels below controls in Carrizo. Moreover, stress imposition also induced higher levels of phosphoenolpyruvate in Carrizo, consistent with increased oxalacetate concentration in this genotype. Nevertheless, stress slightly induced pyruvate accumulation in the two genotypes, although to a higher extent in Cleopatra. Among the TCA cycle intermediates, all stress conditions induced citrate accumulation in Cleopatra but only HS had a similar effect in Carrizo. Similarly, isocitrate accumulated in Carrizo in response to HS. In turn, α-oxoglutarate profiles showed a similar trend to that of citrate: In Carrizo, it responded to high temperatures but stress combination reduced its levels below control values. Contrastingly, Cleopatra exhibited increased levels of α-oxoglutarate in response to WS but even higher levels were found under HS and WS+HS. Levels of succinate also showed contrasting behavior in Carrizo and Cleopatra in response to stress (Figure 3). All stress conditions increased succinate levels in Carrizo especially those involving HS whereas in Cleopatra isolated heat or drought caused decreases in succinate concentration and only stress combination slightly increased its levels. Finally, Cleopatra increased fumarate levels in response to all stress conditions whereas in Carrizo no significant differences were found. Malate levels slightly increased under WS and stress combination in Carrizo but it followed an opposite decreasing trend in Cleopatra under WS.

### Accumulation of metabolites related to the glyoxylate/dicarboxylate pathway

**Figure 4:**
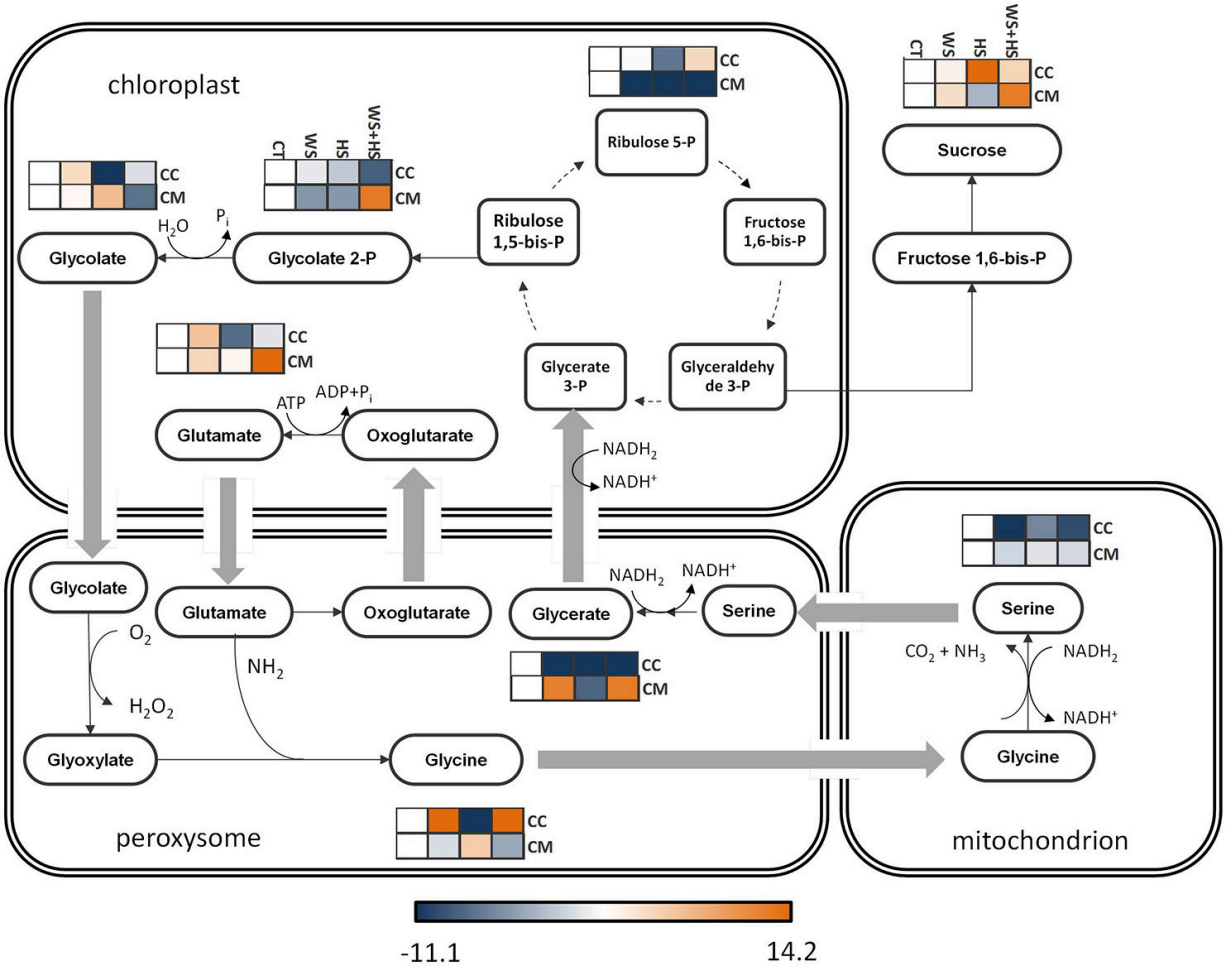
**Levels of metabolites involved in the glyoxylate/dicarboxylate pathway**. Relative levels [expressed as log_2_ (stress/control)] are given besides each detected metabolite as a heatmap: CC, Carrizo and CM, Cleopatra, CT, control; WS, water stress; HS, heat stress; WS+HS, stress combination. Statistical analyses for each metabolite are given in Table S2.

In general, stress induced the accumulation of different intermediates of the chloroplastic phase of the glyoxylate/dicarboxylate cycle in citrus. The accumulation of glycolate 2-P from ribulose-1,5-bis-P was impaired in Carrizo under WS+HS conditions. Nevertheless, glycolate concentration was significantly higher in WS and HS in Carrizo and Cleopatra leaves, respectively. Accumulation profile for the Calvin-cycle intermediate, ribulose 5-P, varied among genotypes. Levels were severely reduced in Cleopatra under all stress conditions whereas they slightly increased in Carrizo in response to WS+HS. Sucrose levels significantly increased in Carrizo in response to all stress conditions, showing the strongest accumulation under HS. In contrast, in Cleopatra, HS had a detrimental effect on sucrose build-up, whereas a moderate induction was observed under WS+HS (Figure 4). Among the peroxysomal metabolites, concentration of glycine followed an opposite trend between the two genotypes: While levels of this aminoacid strongly increased in response to WS and WS+HS in Carrizo leaves, in Cleopatra they decreased under the same stress conditions. Although clear increases in glycine levels were induced in Carrizo by the stress conditions, no parallel effect on serine concentration was observed (Figure 4). Nevertheless, glycerate concentration also differed from that of its precursor serine, showing significant increases in response to WS and WS+HS in Cleopatra and a severe depression in Carrizo in response to all stress conditions.

### Accumulation of metabolites related to phenylpropanoid pathway

**Figure 5:**
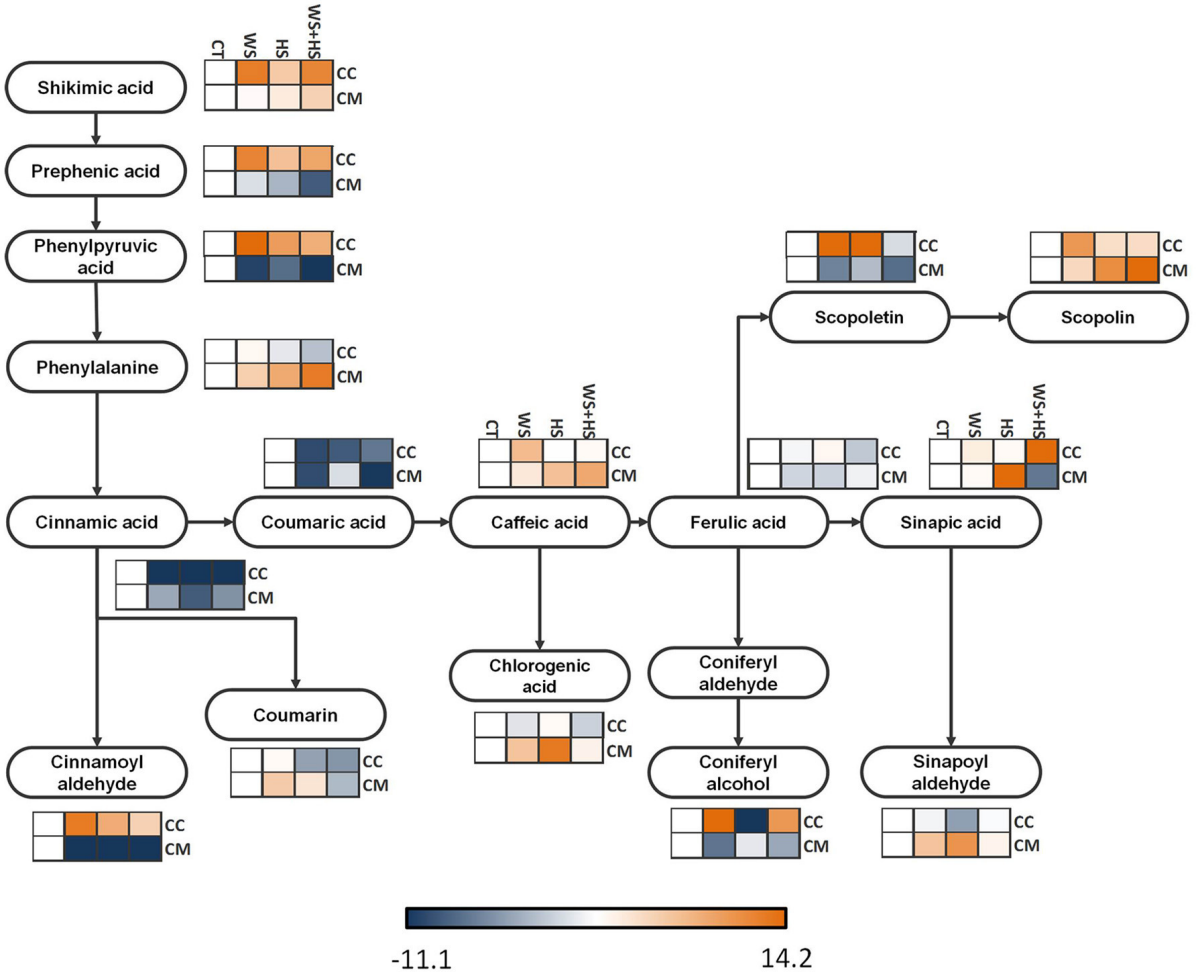
**Levels of metabolites involved in the phenylpropanoid pathway**. Relative levels [expressed as log_2_ (stress/control)] are given besides each detected metabolite as a heatmap: CC, Carrizo and CM, Cleopatra, CT, control; WS, water stress; HS, heat stress; WS+HS, stress combination. Statistical analyses for each metabolite are given in Table S2.

Accumulation of polar metabolites participating in the phenylpropanoid pathway arising from shikimic acid was analyzed, showing different metabolite profiles between genotypes in response to stress: All adverse conditions increased shikimic acid, prephenic acid, and phenylpyruvic acid concentrations in Carrizo whereas levels of these metabolites generally decreased in Cleopatra under the same stress conditions. Nevertheless, phenylalanine concentration increased in Cleopatra in response to WS, HS and especially WS+HS whereas stress treatments had poor impact on this aminoacid in Carrizo (Figure 5). Levels of cinnamic acid, the first phenolic acid product of the reaction catalyzed by the phenylalanine ammonia lyase, were severely reduced after stress imposition, as well as the product of cinnamate hydroxylation, coumaric acid, in leaves of both citrus genotypes. However, cinnamoyl aldehyde accumulated only in Carrizo leaves subjected to WS and into a lesser extent to HS and WS+HS, whereas its levels were severely reduced in Cleopatra. Moreover, caffeic acid levels increased in both genotypes in response to stress following different profiles. In this sense, its levels were higher in Carrizo plants subjected to WS whereas a minor accumulation could be observed upon WS+HS imposition in this genotype. Contrastingly, all stress conditions induced significant increases in the caffeic acid concentration in Cleopatra, although the highest levels were observed in plants subjected to stress combination. Ferulic acid levels did not show any significant alteration in response to stress but, conversely, scopoletin, derived from hydroxylation of feruloyl CoA (Kai et al., 2008), and scopolin, a glycoside of scopoletin, were accumulated in response to all stress conditions in Carrizo and Cleopatra. Moreover, sinapic acid levels also responded positively to WS+HS in Carrizo. However, in Cleopatra, levels of this metabolite increased significantly only in response to HS, whereas stress combination reduced its levels below control values.

Phenolic acids are usually converted into the respective aldehydes via conjugation with coenzyme A and subsequently reduced to alcohols, constituting the building blocks for lignins (Boerjan et al., 2003). Sinapoyl aldehyde exhibited a general increment in response to individual stresses in Cleopatra whereas it was reduced below control levels in Carrizo in response to HS. Coniferyl alcohol (derived from coniferyl aldehyde) accumulated in Carrizo in response to WS and WS+HS and reduced in response to HS. In Cleopatra, levels of this metabolite decreased in response to WS. Coumarin, which is synthesized from cinnamic acid and performs different cell protective roles, was significantly induced by HS in Carrizo and to a lesser extent by WS+HS. Moreover, caffeic acid diverted into the production of chlorogenic acid (Humphreys and Chapple, 2002), showing significantly increased levels in Cleopatra in response to WS and HS. However, levels of this metabolite slightly decreased in Carrizo upon stress imposition (Figure 5).

### Analysis of secondary metabolites by LC-MS

**Figure 6:**
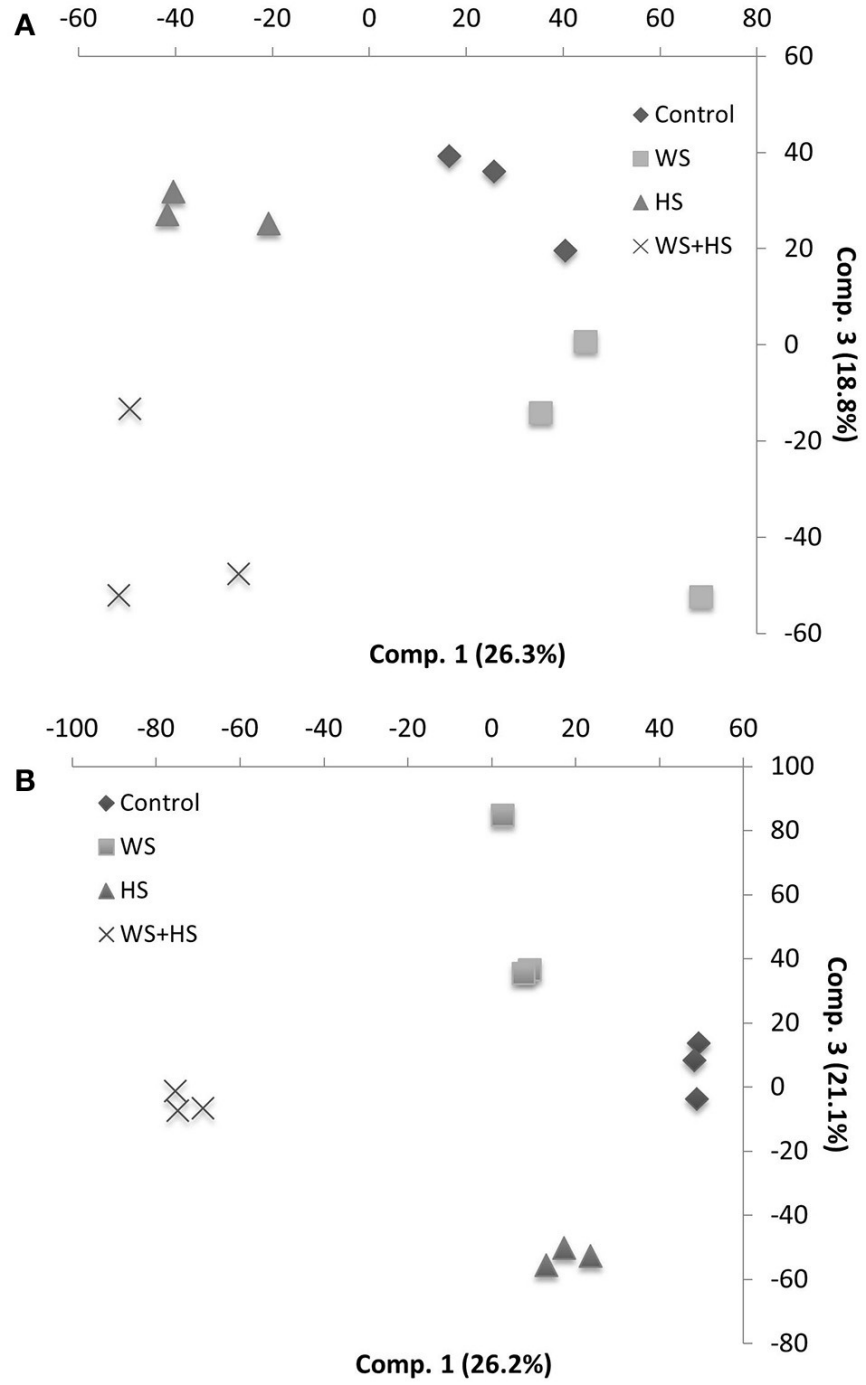
**Partial Least Squares-Discriminant Analysis (PLS-DA) of semi-polar metabolite profiles in Carrizo (A)** and Cleopatra **(B)**.

To determine the accumulation of semi-polar metabolites in leaves of citrus plants subjected to WS, HS and their combination, we performed a LC-MS analysis of compounds extracted from leaves of both citrus plants subjected to the different stresses. Xcms processing of LC-MS metabolite profiles rendered a total of 7195 and 8453 peaks in Carrizo and Cleopatra, respectively, of which 1001 and 2730 had significant variations in area values in the different treatments, as revealed by ANOVA analysis. Partial Least Squares coupled to Discriminant Analysis (PLS-DA) showed a clear impact of heat stress on secondary metabolite composition in Carrizo (26.3% of explained variability) whereas water stress had a lower contribution (18.8%) (Figure 6A). In Cleopatra, however, both heat and water stress had an influence on secondary metabolite composition (21.1%) although the stress combination had a stronger contribution (26.2%) (Figure 6B).

**Figure 7:**
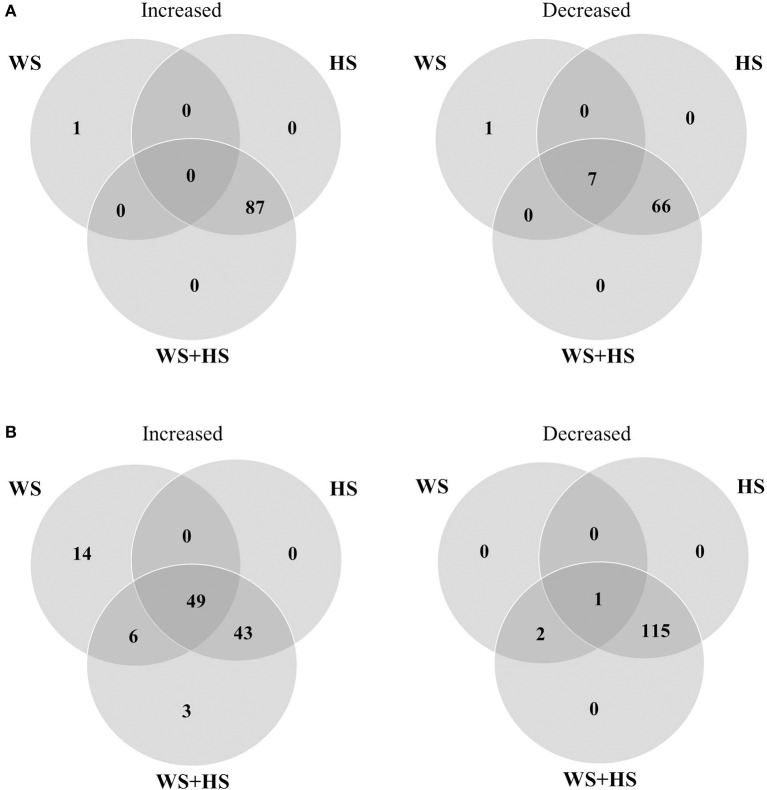
**Venn diagrams depicting overlapping secondary metabolites significantly altered during WS, HS and WS+HS in Carrizo (A)** and Cleopatra **(B)**.

**Figure 8:**
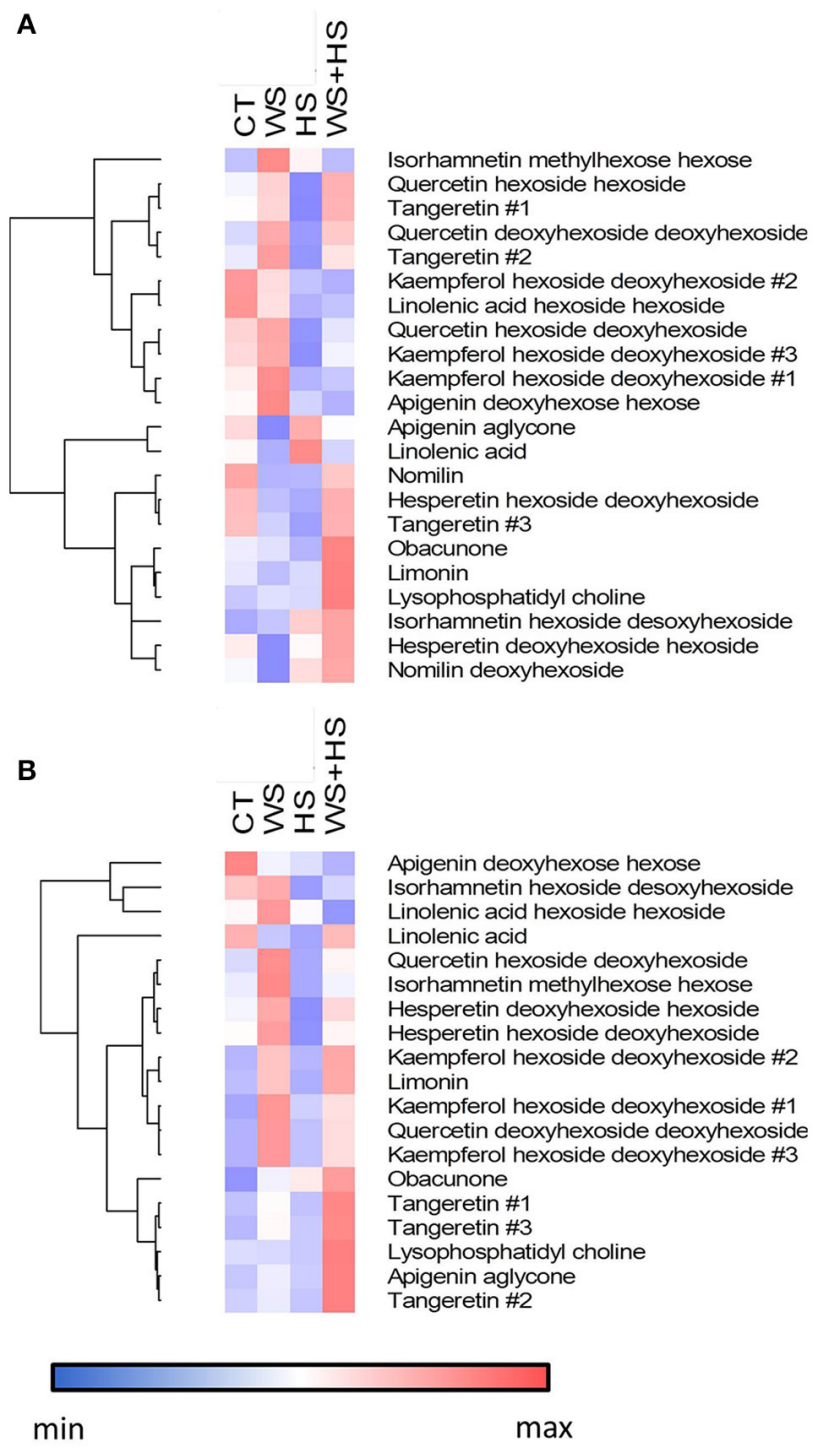
**Hierarchical cluster analysis and heatmap displaying identified secondary metabolites significantly altered in leaves of Carrizo (A)** or Cleopatra **(B)** plants in response to stress.

**Figure 9:**
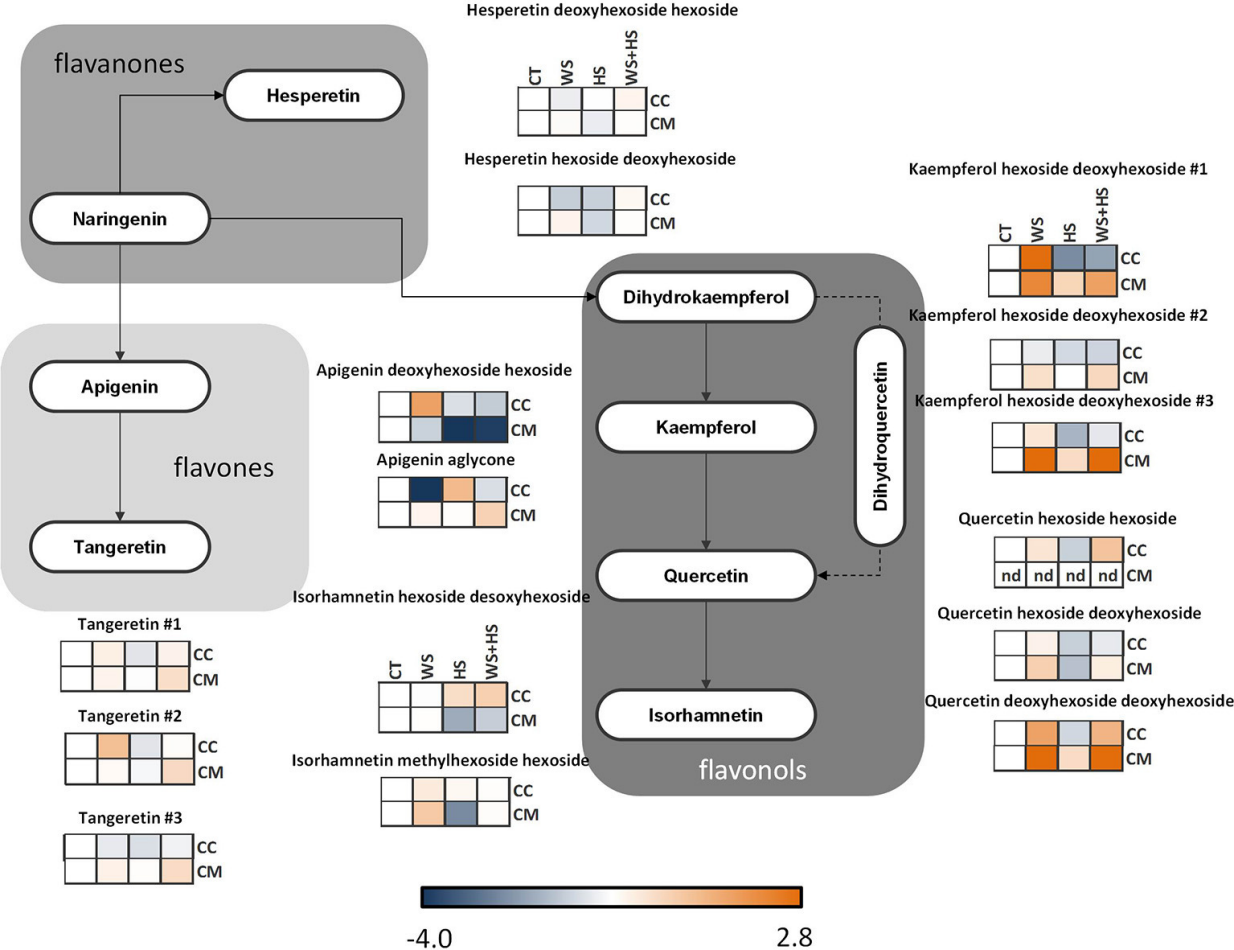
**Levels of metabolites involved in the flavonoid pathway**. Relative levels [expressed as log_2_ (stress/control)] are given besides each detected metabolite as a heatmap: CC, Carrizo and CM, Cleopatra, CT, control; WS, water stress; HS, heat stress; WS+HS, stress combination. Statistical analyses for each metabolite are given in Table S3.

**Figure 10:**
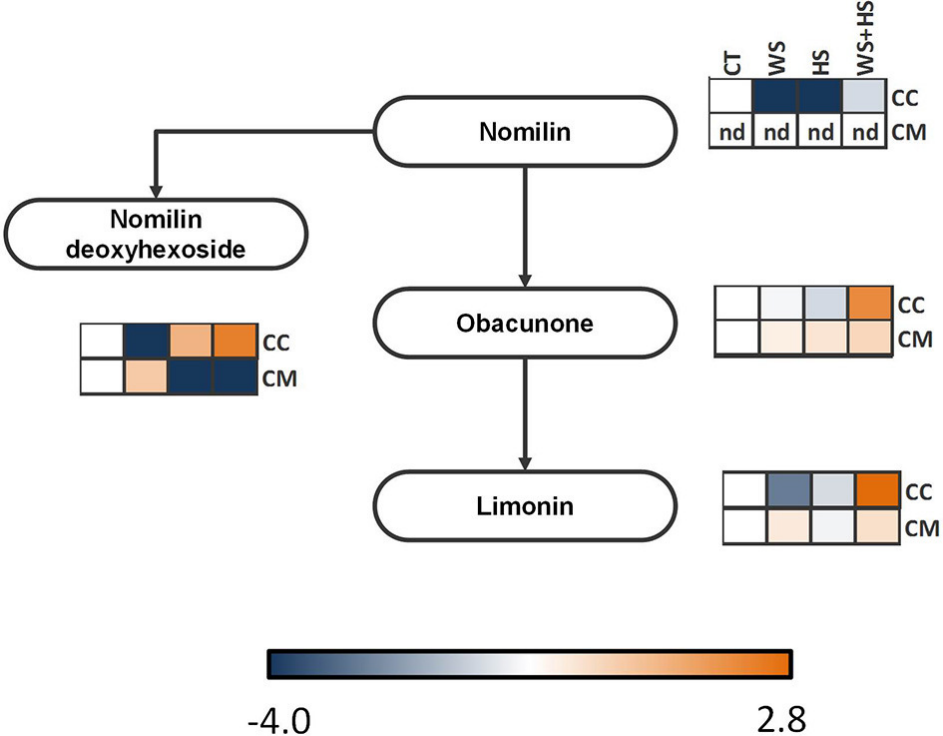
**Levels of metabolites involved in the limonoid pathway**. Relative levels [expressed as log_2_ (stress/control)] are given besides each detected metabolite as a heatmap: CC, Carrizo and CM, Cleopatra, CT, control; WS, water stress; HS, heat stress; WS+HS, stress combination. Statistical analyses for each metabolite are given in Table S3.

The specific profile for each stress treatment as well as the metabolite overlapping among treatments was studied and results are depicted as Venn diagrams in Figure 7. Carrizo leaves displayed a clear response to HS applied individually or in combination with WS. In this sense, levels of 87 and 66 metabolites increased and decreased, respectively, during HS and WS+HS, likely as a result of the incidence of HS (Figure 7A). Identification of metabolites is shown in Table 1. Among them, we found significantly altered flavonoids such as isorhamnetin hexoside desoxyhexoside, limonoids (nomilin deoxyhexoside), or the phospholipid derivative lysophosphatidyl choline (Table 1 and Figures 8–10 and Figure S1). Furthermore, we also found that levels of several compounds decreased in response to HS and WS+HS in Carrizo leaves (Figures 8, 9 and Figure S1) including flavonoids like kaempferol derivatives or apigenin deoxyhexose hexose. On the other hand, as indicated also by PLS-DA (Figure 6B), we observed a significant response of Cleopatra to WS, finding 14 secondary metabolites whose levels were significantly altered during WS applied individually, including flavonoids (quercetin hexoside deoxyhexoside and hesperetin hexoside deoxyhexoside), limonoids (nomilin deoxyhexoside), or lipid derivatives such as linolenic acid hexoside hexoside (Figures 8, 9 and Figure S1). Additionally, 49 compounds were accumulated in response to all stress treatments (Figure 7B), including those depicted in Figure 8, of which 43 were specifically accumulated in response to HS and WS+HS in Cleopatra leaves. Moreover, levels of 6 secondary metabolites were altered in Cleopatra plants in response to WS and WS+HS and only 3 compounds were found to be significantly accumulated in response to WS+HS. Moreover, a total of 115 metabolites specifically decreased in Cleopatra leaves subjected to HS and WS+HS. Only levels of one compound decreased in response to all stress treatments and 2 metabolites also decreased in Cleopatra plants in response to WS and WS+HS (Figure 7B).

**Table 1:** **Identification of compounds analyzed by LC-MS**.

**Metabolite**	**m/z (ESI+)**	**Adduct**	**m/z (ESI-)**	**Adduct**	**Rt (min)**	**Rt (s)**
**FLAVONOIDS**						
Quercetin hexoside hexoside	627.16	[M+H]^+^	625.14	[M-H]^−^	5.50	330
	465.11	[M-hexose]^+^				
	303.05	[M-2hexose]^+^				
Quercetin hexoside deoxyhexoside	609.15	[M+H]^+^	607.14	[M-H]^−^	6.00	360
	303.06	[M-hexose-deoxyhexose]^+^				
	449.10	[M-hexose]^+^				
Quercetin deoxyhexoside deoxyhexoside	595.17	[M+H]^+^	593.16	[M-H]^−^	6.34	380.4
	449.12	[M-deoxyhexose]^+^				
	303.07	[M-deoxyhexose]^+^				
Hesperetin deoxyhexoside hexoside	303.09	[M-deoxyhexose]^+^	609.18	[M-H]^−^	6.60	396
	611.19	[M+H]^+^	301.08	[M-deoxyhexose-hexose]^−^		
	449.15	[M-hexose]^+^				
Hesperetin hexoside deoxyhexoside	611.20	[M+H]^+^	609.18	[M-H]^−^	6.77	406.2
	449.15	[M-hexose]^+^	301.08	[M-hexose-deoxyhexose]^−^		
	303.09	[M-deoxyhexose]^+^	447.14	[M-hexose]^−^		
Kaempferol hexoside deoxyhexoside #1	287.07	[M-deoxyhexose-hexose]^+^	285.06	[M-deoxyhexose-hexose]^−^	5.99	359.4
	595.16		593.15	[M-H]^−^		
	449.10		447.09	[M-deoxyhexose]^−^		
Kaempferol hexoside deoxyhexoside #2	595.16	[M+H]^+^	593.16	[M-H]^−^	6.13	367.8
	287.07	[M-hexose-deoxyhexose]^+^	285.06	[M-hexose-deoxyhexose]^−^		
			447.11	[M-deoxyhexose]^−^		
Kaempferol hexoside deoxyhexoside #3	595.16	[M+H]^+^	593.16	[M-H]^−^	6.35	381
	449.12	[M-hexose]^+^	285.06	[M-hexose-deoxyhexose]^−^		
	287.07	[M-hexose-deoxyhexose]^+^				
Isorhamnetin hexoside desoxyhexoside	625.18	[M+H]^+^	623.17	[M-H]^−^	6.40	384
	317.07	[M-deoxyhexose-hexose]^+^	315.07	[M-deoxyhexose-hexose]^−^		
	479.13	[M-deoxyhexose^]+^				
Isorhamnetin methylhexose hexose	623.16	[M+H]^+^	621.15	[M+H]^−^	6.90	414
	317.07	[M-methylhexose-hexose]^+^	315.06	[M-methylhexose-hexose]^−^		
Apigenin deoxyhexose hexose	271.06	[M-deoxyhexose-hexose]^+^	269.05	[M-deoxyhexose-hexose]^−^	6.45	387
	579.17	[M+H]^+^	577.16	[M-H]^−^		
	433.11	[M-deoxyhexose]^+^				
						
Apigenin aglycone	271.06	[M+H]^+^	269.06	[M-H]^−^	8.66	519.6
Tangeretin #1	373.14	[M+H]^+^	–		9.40	564
Tangeretin #2	373.14	[M+H]^+^	–		9.90	594
Tangeretin #3	373.14	[M+H]^+^	–		11.20	672
**PHENYLPROPANOIDS**						
Scopolin	193.07	[M-hexose]^+^	–		4.90	294
	355.11	[M+H]^+^				
Scopoletin	193.07	[M+H]^+^	–		5.80	348
**TRITERPENOIDS**						
Nomilin deoxyhexoside	515.22	[M-deoxyhexose]^+^	531.22	[M-deoxyhexose+H_2_O]^−^	7.90	474
	487.23	[M-CO]^+^				
	469.22	[M-CH_2_O_2_]^+^				
	455.21	[M-C_2_H_4_O_2_]^+^				
	413.20	[M-C_4_H_6_O_3_]^+^				
	661.27	[M+H]^+^				
Nomilin	515.22	[M+H]^+^	453.19	[M-C_2_H_3_O_2_]^−^	10.50	630
	487.23	[M-CO]^+^	513.22	[M-H]^−^		
			425.20	[M-CO]^−^		
Obacunone	455.21	[M+H]^+^	453.20	[M-H]^−^	11.10	666
	411.17	[M-C_2_H_4_O]^+^				
Limonin	471.20	[M+H]^+^	469.19	[M-H]^−^	9.90	594
	443.21	[M-CO]^+^				
	425.20	[M-H_2_O]^+^				
**LIPIDS AND DERIVATIVES**						
Lysophosphatidyl choline	496.34	[M-C_18_H_32_O]^+^	–		13.80	828
	263.24	[M-C_24_H_49_NO_7_P]^+^				
Linolenic acid	279.23	[M+H]^+^	277.22	[M-H]^−^	12.30	738
Linolenic acid hexoside hexoside	353.27	[M-hexose+H_2_O]^+^	277.22	[M-hexose-hexose]^+^	11.57	694.2
	497.31	[M-hexose]^+^	657.35	[M-H]^−^		
	261.22	[M-hexose-hexose-H_2_O]^+^				
	659.36	[M+H]^+^				
	335.26	[M-hexose]^+^				

*Ordering of m/z values reflects their relative abundance*.

### Flavonoid-related metabolites

The accumulation profile of different flavonoid-related compounds in leaves of Carrizo and Cleopatra subjected to WS, HS, and WS+HS was also studied. As shown in Figure 9, different patterns of flavonoid accumulation were observed between both citrus genotypes and also among stress treatments. Therefore, levels of flavones such as the different tangeretin isomers were constitutively higher in Cleopatra than in Carrizo (Figure S1) but WS significantly increased their levels in Carrizo. However, in Cleopatra, WS+HS induced the accumulation of these compounds. Flavones, such as apigenin and its derivatives, showed an opposite constitutive trend: While apigenin aglycone levels were much higher in Cleopatra apigenin deoxyhexoside hexoside levels were higher in Carrizo (Figure S1). In turn, apigenin deoxyhexoside hexoside showed a significant accumulation in response to WS in Carrizo whereas it was slightly reduced below control levels in Cleopatra under HS and WS+HS (Figure 8). Meanwhile, apigenin aglycone levels were significantly increased in response to WS+HS in Cleopatra whereas in Carrizo, the levels of this metabolite were reduced with respect to control values during WS. Stress treatments had no impact on the accumulation of the different hesperetin derivatives (Figure 9, Figure S1 and Table S3) in both citrus genotypes. However, hesperetin derivatives were constitutively higher in Cleopatra than in Carrizo (Figure S1). Furthermore, individual and combined stress conditions induced the accumulation of kaempferol derivatives in Cleopatra with almost identical profiles: WS and WS+HS showing the highest levels (Figure 9). In addition, levels of kaempferol hexoside deoxyhexoside #2 were in general much higher in Cleopatra than in Carrizo (Figure S1). Regarding quercetin derivatives, the effect of stress treatments was similar to that of kaempferol derivatives (except for quercetin hexoside hexoside that was not detected in Cleopatra), showing a significant accumulation in response to WS and WS+H in Cleopatra. On the other hand, no impact on the accumulation of isorhamnetin-related compounds was observed in Carrizo leaves in response to stress. Moreover, constitutive levels of isorhamnetin hexoside deoxyhexoside were higher in Cleopatra and those of isorhamnetin methylhexose hexose higher in Carrizo (Figure S1). On the other hand, in Cleopatra, levels of isorhamnetin hexoside desoxyhexoside decreased in response to HS whereas isorhamnetin methylhexose hexose was accumulated under WS in this genotype.

### Limonoid-related metabolites

Limonoids are naturally-occurring triterpenoids in rutaceae whose accumulation or depletion in response to individual and combined stress conditions was evaluated (Figure 10). Nomilin was not detected in Cleopatra leaves while in Carrizo the levels of this metabolite were reduced in response to individual WS and HS. Moreover, the accumulation of nomilin deoxyhexoside followed opposite patterns in both citrus genotypes: While Carrizo decreased the concentration of this metabolite under WS and accumulated it under HS and WS+HS, Cleopatra showed significantly increased levels under WS but reduced under HS and WS+HS. Obacunone and limonin levels increased in Cleopatra plants in response to WS and WS+HS whereas in Carrizo, the accumulation of both metabolites was especially marked in response to WS+HS (Figure 10).

### Other lipid-related compounds

Constitutive levels of linolenic acid, linolenic acid dihexoside, and lysophosphatidyl choline were higher in Cleopatra than in Carrizo and, in addition, showed different regulation patterns in the two genotypes (Figure 8 and Figure S1). Lysophosphatidyl choline levels increased mainly in response to WS+HS in both genotypes although to a higher extent in the sensitive genotype Cleopatra. Conversely, levels of linolenic acid dihexoside decreased in response to WS+HS and increased under WS conditions in Cleopatra. In Carrizo, however, HS and WS+HS reduced levels of linolenic acid dihexoside (Figure 8 and Figure S1). Finally, stress had little impact on linolenic acid levels, showing slight reductions in response to WS and WS+HS in Carrizo and in response to WS and HS in Cleopatra.

## Discussion

In natural environments, plants have to face different combinations of abiotic stresses, tailoring specific physiological and molecular responses to deal with a new stress situation, different from the individual stresses (Mittler and Blumwald, 2010; Suzuki et al., 2014; De Boeck et al., 2015; Hu et al., 2015; Liu et al., 2015; Zhang et al., 2015).

We recently conducted a study focusing on the physiological and hormonal responses of two citrus genotypes, Carrizo citrange and C. mandarin, to a combination of drought and high temperatures. Data demonstrated that Carrizo is more tolerant than Cleopatra to heat applied individually or in combination with drought, concluding that higher transpiration and photosynthetic rates along with a stronger antioxidant defense is crucial in determining tolerance to a combination of drought and heat stress in citrus (Table S1; Zandalinas et al., 2016b). However, it has been previously shown that citrus respond to environmental stresses by modifying their metabolism (Tanou et al., 2014; Ziogas et al., 2015; Shiratake and Suzuki, 2016). Therefore, a metabolomics approach was followed to identify additional metabolic mechanisms underlying the superior tolerance of Carrizo to HS and WS+HS.

In the present work, polar and semi-polar metabolites differentially altered during each stress condition were identified. Cleopatra and Carrizo leaves showed a similar number of altered polar primary compounds spanning different metabolic pathways. However, in Cleopatra, a large number of polar metabolites were significantly altered in response to WS+HS (Figure 2). Therefore, a total of 68 primary compounds increased their concentration in leaves of Cleopatra seedlings subjected to stress, of which 48 responded to WS+HS and only 1 to WS. In contrast, only 8 polar metabolites were accumulated in Carrizo leaves in response to stress combination. These data suggest that under the same stress conditions, the studied citrus genotypes alter their metabolism in different manners leading to different polar metabolite profiles.

The energy pathways glycolysis, TCA cycle and the mitochondrial electron transport chain are essential for ATP and NADH provision in heterotrophic cells, but also have a wide range of other physiological functions (Fernie et al., 2004). In our work, some metabolites involved in TCA cycle accumulated in Cleopatra in response to stress, including α-oxoglutarate, fumarate, and citrate (Figure 3). In addition, Cleopatra leaves seemed to notably accumulate some glycolysis intermediates such as glucose-6-P, glyceraldehyde-3-P, glycerate-2-P, and pyruvate upon stress imposition. Some of these intermediates, involved in glycolysis and TCA cycle (fructose, pyruvate, and α-oxoglutarate), were accumulated in an additive manner in Cleopatra indicating that the combination of WS and HS modifies the profile of glycolytic and TCA cycle metabolites in a way paralleling the higher sensitivity of Cleopatra to drought and heat combination (Zandalinas et al., 2016b). In our previous work, Cleopatra seedlings closed stomata during WS+HS limiting CO_2_ fixation (Table S1; Zandalinas et al., 2016b). In the present work, results showed that metabolites involved in alternative pathways such as TCA cycle and glycolysis were accumulated in Cleopatra (Figure 3), suggesting their activation to supply energy demand and metabolic intermediates.

The glyoxylate/dicarboxylate pathway is involved in the dissipation of excess reducing equivalents as well as energy that could result in ROS production (Voss et al., 2013). In Carrizo plants, we observed an increased accumulation of sucrose in response to HS in contrast to Cleopatra. The higher photosynthetic rate of Carrizo compared to Cleopatra under HS (Table S1; Zandalinas et al., 2016b) could be associated to a significant higher sucrose content. Although no data on starch content has been provided, it could be speculated that higher sucrose availability is rather a result of transport of carbohydrates derived from CO_2_ fixation. To this respect, data presented here suggest that sucrose biosynthesis derived from the higher CO_2_ fixation rate in Carrizo, could constitute an advantage under stressful conditions possibly serving as an energy source for non-photosynthetic tissues or being stored as starch (Figure 4).

Phenylpropanoid pathway intermediates represent a major flow of carbon from primary metabolism into secondary metabolism. Among these metabolites, the coumarin scopoletin, derived from feruloyl-CoA, is converted into scopolin by glycosylation, and both metabolites are likely to be involved in plant responses to stress (Kai et al., 2008). Our results suggest that Cleopatra plants accumulated scopolin and scopoletin under WS and WS+HS whereas Carrizo accumulated both metabolites in response to both stresses when acting isolated from each other as well to WS+HS. These results suggest that scopoletin and scopolin would protect citrus plants from WS and WS+HS. In addition, whereas only Carrizo accumulated these compounds in response to HS, Cleopatra plants subjected to HS accumulated sinapic acid. Moreover, in response to stress, Carrizo accumulated coniferyl alcohol derived from coniferyl aldehyde, as well as cinnamoyl aldehyde (Figure 5), precursors of lignins, suggesting that Carrizo plants could induce lignin production and deposition in cell walls to protect plant cells from abiotic stress.

On the whole, our data indicate that Cleopatra, as a sensitive genotype to HS and WS+HS, deeply alters the accumulation of polar metabolites, especially those supporting cellular metabolite and energy requirements, in order to face the damaging effects caused by stress combination. On the other hand, Carrizo showed modifications in the levels of polar metabolites oriented to generate metabolite intermediates for carbohydrate, phenylpropanoid and lignin biosynthesis. Plant secondary metabolites are often referred to as compounds that have no fundamental role in the maintenance of life processes in plants, but they are important in the interaction with the surrounding environment. In this sense, different stresses regulate the production of secondary metabolites, which are most often involved in plant defense and stress acclimation (Zhao et al., 2005).

After studying the different accumulation of polar metabolites related to plant primary metabolism, we investigated whether secondary metabolites could play an important role in the tolerance of citrus plants to WS, HS, and WS+HS. In general terms, alteration in levels of secondary metabolites overlapped between HS and WS+HS (Figure 7). Among them, some flavonoids and limonoids were differently altered during WS and HS applied alone or in combination in both citrus genotypes (Figures 9, 10).

Although the specific physiological role of flavonoids in citrus remains largely unknown, recent studies in citrus juices have identified the main flavonoids and derivatives of chemotaxonomic significance (Arbona et al., 2015) and with high radical scavenging activity (Patil et al., 2009). In general, flavanones and flavones are poorly represented in plants, except in citrus. Actually, flavanones, along with flavones and flavonols, are one of the main secondary metabolites found in citrus juices (Djoukeng et al., 2008; Arbona et al., 2015). In addition, naringenin and hesperetin are considered the most abundant flavanones mainly occurring as glycoside derivatives. In citrus tissues, flavones are presented as glycoside derivatives, and flavonols, synthesized from flavanones by hydroxylation, include kaempferol, quercetin, and isorhamnetin (Carisiti et al., 2006; Djoukeng et al., 2008; Arbona et al., 2015). In our work, we could identify several compounds involved in the biosynthesis of flavonoids starting from naringenin (Figure 9, Figure S1 and Table 1), finding important differences between citrus genotypes subjected to WS and HS isolated from each other or in combination. Stress imposition had a poor impact in flavanone accumulation but flavonols were greatly induced in Cleopatra seedlings. In general, stress had a significant impact on flavonol accumulation in Cleopatra leaves. In line with our previous report (Zandalinas et al., 2016b), the stronger flavonol accumulation observed in Cleopatra could be related to its higher sensitivity to stress. Hence, accumulation of these flavonoids could constitute a photoprotective response in leaves of sensitive Cleopatra. Moreover, flavones derived from naringenin were accumulated differently in both genotypes in response to stress treatments being apigenin and polymethoxylated flavones accumulated in Cleopatra in response to WS+HS. These polymethoxylated flavones could be involved in maintaining a high antioxidant activity under stress as previously shown (Yu et al., 2005). These differences in flavone content are consistent with previous data (Zandalinas et al., 2016b), in which higher oxidative damage was observed in Cleopatra during WS+HS, requiring thus an increased antioxidant response respect to Carrizo.

Limonoids are highly oxygenated triterpenes present in Rutaceae and Meliaceae (Hasegawa et al., 1980) that have been reported to exhibit a role in ROS detoxification through the induction of glutathione S-transferase (GST) activity (Yu et al., 2005; Perez et al., 2009). Our results showed an accumulation of obacunone and limonin in Cleopatra in response to stress treatments whereas nomilin could not be detected in this genotype (Figure 10). On the contrary, in Carrizo, levels of nomilin glycoside increased in response to HS and WS+HS, as well as obacunone and limonin content under WS+HS conditions. Interestingly, the accumulation of fatty acids and derivatives, such as lysophosphatidyl choline under WS+HS conditions increased. Lysophospholipids are hydrolyzed from plasma membrane lipids and act as signaling molecules (Hou et al., 2016). Alterations in lipid profile could be associated to an altered metabolism oriented to modify plasma membrane composition under high temperature conditions. Nevertheless, linolenic acid levels were less affected by stress than the corresponding dihexoside or the membrane lipid-derived molecule lysophosphatidyl choline.

Results presented here suggest that the different profile in semi-polar metabolite accumulation under different abiotic conditions could reflect the contrasting ability of Carrizo and Cleopatra plants to tolerate stress treatments. Along with induction of several secondary metabolites, rise in tryptophan, precursor of the auxin indole 3-acetic acid and different indolic compounds, accompanied stress imposition in both citrus genotypes. Moreover, increased basal tyrosine levels correlated with the higher flavonoid concentration found in Cleopatra and could also account for the greater induction of these metabolites in this genotype (Figure S2). It has been previously reported that *A. thaliana* and *Thellungiella halophila* exhibited different regulation of secondary metabolism with a low degree of overlap among them when subjected to identical stress conditions (Arbona et al., 2010). In addition, Morsy et al. (2007) found that two rice genotypes with contrasting abiotic stress tolerance, showed different patterns of metabolite accumulation under low temperature, salt and osmotic stress related to their respective ability to tolerate abiotic stress. Results presented here expand these previous findings, since different profiles of secondary metabolite (Figures 8–10) as well as some amino acids involved in secondary metabolism (Figure S2) were observed in the two citrus genotypes under drought and heat applied individually or in combination. As previously suggested, this different metabolism in citrus under adverse environmental conditions could be a result of the specificity of the secondary metabolism and also the different stress tolerance of Carrizo and Cleopatra plants (Zandalinas et al., 2016b).

On the whole, the different tolerance response of the two citrus genotypes studied could be explained by the particular basal metabolism as well as its different regulation. In this sense, it has been previously reported in two durum wheat cultivars differing in water use efficiency that stress combination could either lead to an enhanced or reduced gene expression respect to that observed under isolated stresses in the sensitive and the tolerant genotype, respectively (Aprile et al., 2013). Therefore, the higher capability of Carrizo to cope with stress-induced oxidative damage (Arbona et al., 2008) and the particular adjustment of transpiration rate during WS+HS (Zandalinas et al., 2016b) evidence the increased tolerance of Carrizo plants to WS+HS with respect to Cleopatra. In addition, this higher ability of Carrizo to deal with WS+HS could prevent further modification of its metabolism. Conversely, due to its high sensitivity, Cleopatra requires a deep alteration of its primary and secondary metabolism in order to cope with stress-induced physiological and biochemical imbalances.

## Author contributions

SZ, VA, and AG designed and supervised the research. SZ and VA performed the research. SZ and VA wrote the first draft of the manuscript and prepared figures. SZ, CS, JB, AG, and VA revised subsequent versions of the manuscript and prepared the final version. All authors have read and approved the final version of the manuscript.

## Funding

This work was supported by Ministerio de Economía y Competitividad (MINECO) [AGL2013-42038-R, AGL2016-76574-R] and Universitat Jaume I [UJI-B2016-23/UJI-B2016-24]. SZ was supported by a predoctoral fellowship from Universitat Jaume I.

### Conflict of interest statement

The authors declare that the research was conducted in the absence of any commercial or financial relationships that could be construed as a potential conflict of interest.
