# Supplementary material for: Activation of Secondary Metabolism in Citrus Plants Is Associated to Sensitivity to Combined Drought and High Temperatures
Source: Front Plant Sci. 2017 Jan 9;7:1954. doi: 10.3389/fpls.2016.01954 (PMC5220112; doi:10.3389/fpls.2016.01954)
Supplement: Supplementary file 1 [file Table1.DOCX]

| CT | | WS | | HS | | WS+HS | | p-value | | |  |
| --- | --- | --- | --- | --- | --- | --- | --- | --- | --- | --- | --- |
| Mean±SD | | **Mean±SD** | | **Mean±SD** | | **Mean±SD** | | **S** | **G** | **SxG** |  |
| A |  |  |  |  |  |  |  |  |  |  |  |
| Carrizo | 6.92±0.50 C | | 1.54±0.73 B | | 9.27±0.89 E | | 1.61±0.14 B | | *** | *** | * |
| Cleopatra | 5.40±0.32 C | | 0.42±0.11 AB | | 5.66±0.28 C | | 0.24±0.08 A | |  |  |  |
| gs |  |  |  |  |  |  |  |  |  |  |  |
| Carrizo | 30.00±2.25 B | | 10.00±1.33 A | | 100.00±3.96 C | | 10.00±1.41 A | | *** | *** | *** |
| Cleopatra | 30.00±2.91 B | | 5.00±1.00 A | | 40.00±1.33 B | | 10.00±1.00 A | |  |  |  |
| E |  |  |  |  |  |  |  |  |  |  |  |
| Carrizo | 0.92±0.03 C | | 0.23±0.09 AB | | 4.61±0.24 E | | 0.94±0.14 C | | *** | *** | *** |
| Cleopatra | 0.88±0.06 BC | | 0.14±0.01 A | | 2.51±0.06 A D | | 0.40±0.01 AB | |  |  |  |
| ci/ca |  |  |  |  |  |  |  |  |  |  |  |
| Carrizo | 0.30±0.02 A | | 0.32±0.05 A | | 0.50±0.03 BC | | 0.49±0.04 BC | | ** | ** | * |
| Cleopatra | 0.46±0.02 AB | | 0.58±0.06 C | | 0.45±0.02 AB | | 0.83±0.04 D | |  |  |  |
| Φ_PSII_ |  |  |  |  |  |  |  |  |  |  |  |
| Carrizo | 0.75±0.01 C | | 0.69±0.01 B | | 0.78±0.03 C | | 0.63±0.02 A | | *** | *** | *** |
| Cleopatra | 0.74±0.01 C | | 0.70±0.00 B | | 0.63±0.02 A | | 0.60±0.02 A | |  |  |  |
| Fv/Fm |  |  |  |  |  |  |  |  |  |  |  |
| Carrizo | 0.81±0.02 C | | 0.80±0.01 CB | | 0.81±0.01 C | | 0.77±0.02 AB | | ** | *** | ** |
| Cleopatra | 0.81±0.01 C | | 0.80±0.01 CB | | 0.76±0.02 A | | 0.73±0.01 A | |  |  |  |

**Table S1**. Physiological parameters of Carrizo and Cleopatra plants in response to drought (WS), heat stress (HS) and the combination of drought and heat stress (WS+HS). Photosynthetic rate, A; stomatal conductance, gs (in mmol m^-1^ s^-2^); transpiration rate, E; carboxylative efficiency, ci/ca; quantum efficiency of PSII, Φ_PSII_; maximum efficiency of PSII, Fv/Fv. Different letters denote statistical significance at p≤0.05. S: stress treatment; G: genotypes; SxG: interaction stress treatment x genotype. *p<0.05; **p<0.01; ***p<0.001; ns: no statistical differences.
