# Supplementary material for: Activation of Secondary Metabolism in Citrus Plants Is Associated to Sensitivity to Combined Drought and High Temperatures
Source: Front Plant Sci. 2017 Jan 9;7:1954. doi: 10.3389/fpls.2016.01954 (PMC5220112; doi:10.3389/fpls.2016.01954)
Supplement: Supplementary file 2 [file Table2.DOCX]

| Compound | Carrizo | | | | Cleopatra | | | | p-value | | |
| --- | --- | --- | --- | --- | --- | --- | --- | --- | --- | --- | --- |
|  | CT | WS | HS | WS+HS | CT | WS | HS | WS+HS | S | G | SxG |
| Caffeic acid | AB | B | AB | AB | AB | AB | AB | B | * | * | ns |
| Chlorogenic acid | A | A | A | A | A | AB | B | A | ns | ** | ns |
| Cinnamic acid | A | A | A | A | A | A | A | A | ns | ns | ns |
| Cinnamoyl aldehyde | AB | B | B | AB | AB | A | A | A | ns | ** | ns |
| Citrate | A | A | A | A | A | A | B | B | * | ** | * |
| Coniferyl alcohol | AB | BC | A | B | AB | A | A | A | * | * | ns |
| Coumaric acid | A | A | A | A | A | A | A | A | ns | * | ns |
| Coumarin | A | AB | C | BC | A | A | A | A | * | *** | * |
| Ferulic acid | A | A | A | A | A | A | A | A | ns | ns | ns |
| Dihydroxyacetone P | AB | BC | AB | B | AB | AB | A | AB | * | * | ns |
| Fructose | A | AB | B | B | A | A | B | B | ** | ns | ns |
| Fructose 6-P | AB | AB | AB | AB | AB | A | AB | A | * | ns | ns |
| Fumarate | AB | AB | AB | AB | AB | B | AB | B | ns | ** | ns |
| Glucose | A | A | A | A | - | - | - | - | ns | - | - |
| Glucose 6-P | A | A | A | A | A | AB | A | A | * | ns | ns |
| Glutamate | AB | AB | A | AB | AB | AB | AB | B | * | * | ns |
| Glyceraldehyde 3-P | AB | A | A | A | AB | B | B | AB | * | ** | ** |
| Glycerate | AB | A | A | A | AB | B | A | B | ns | ** | ns |
| Glycerate 2-P | AB | AB | B | A | AB | B | B | AB | * | ** | * |
| Glycerate 3-P | AB | B | AB | A | AB | A | A | AB | * | * | ns |
| Glycine | AB | BC | A | BC | AB | AB | B | AB | *** | *** | *** |
| Glycolate | AB | B | A | AB | AB | AB | B | A | * | * | ns |
| Glycolate 2-P | AB | AB | AB | A | AB | A | A | B | * | * | ns |
| Isocitrate | AB | AB | A | A | AB | AB | B | AB | ns | * | * |
| Malate | AB | AB | AB | AB | AB | A | A | A | ns | ** | ns |
| Oxalacetate | A | AB | A | B | A | A | AB | A | * | ** | ns |
| Phenylalanine | A | A | A | A | A | AB | AB | B | ns | ** | ns |
| Phenylpyruvic acid | C | ABC | BC | ABC | A | AB | A | AB | ns | *** | ns |
| Phosphoenolpyruvate | C | ABC | BC | ABC | A | AB | AB | AB | ns | *** | ns |
| Prephenic acid | AB | B | AB | AB | AB | A | A | A | * | ** | ns |
| Pyruvate | A | AB | AB | AB | A | A | AB | B | * | ns | ns |
| Ribulose 5-P | AB | AB | A | B | AB | A | A | A | ns | * | ns |
| Scopoletin | A | ABC | AB | A | E | CDE | DE | BCD | ns | *** | ** |
| Scopolin | AB | B | AB | AB | A | AB | AB | C | *** | ns | *** |
| Serine | AB | A | A | A | AB | AB | AB | AB | ns | * | ns |
| Shikimic acid | A | B | AB | B | A | A | A | A | * | ** | ns |
| Sinapic acid | AB | AB | AB | BC | AB | AB | B | A | * | * | ns |
| Sinapoyl aldehyde | AB | AB | A | AB | AB | AB | B | AB | ns | * | ns |
| Succinate | AB | BC | ABC | C | ABC | A | A | A | ns | *** | * |
| Sucrose | AB | AB | C | AB | AB | AB | A | B | ** | * | * |
| α-oxoglutarate | AB | B | B | A | AB | B | B | C | ** | *** | ** |

**Table S2**. Analysis of variance of polar metabolite levels in Carrizo and Cleopatra plants in response to drought (WS), heat stress (HS) and the combination of drought and heat stress (WS+HS). Different letters denote statistical significance at p≤0.05. S: stress treatment; G: genotypes; SxG: interaction stress treatment x genotype. *p<0.05; **p<0.01; ***p<0.001; ns: no statistical differences.
