# Supplementary material for: Activation of Secondary Metabolism in Citrus Plants Is Associated to Sensitivity to Combined Drought and High Temperatures
Source: Front Plant Sci. 2017 Jan 9;7:1954. doi: 10.3389/fpls.2016.01954 (PMC5220112; doi:10.3389/fpls.2016.01954)
Supplement: Supplementary file 3 [file Table3.DOCX]

| **Compound** | **Carrizo** | | | | **Cleopatra** | | | | **p-value** | | |
| --- | --- | --- | --- | --- | --- | --- | --- | --- | --- | --- | --- |
|  | **CT** | **WS** | **HS** | **WS+HS** | **CT** | **WS** | **HS** | **WS+HS** | **S** | **G** | **SxG** |
| Quercetin hexoside hexoside | A | A | A | A | - | - | - | - | ns | - | - |
| Quercetin hexoside deoxyhexoside | B | B | AB | AB | AB | B | A | AB | * | ** | ns |
| Quercetin deoxyhexoside deoxyhexoside | ABC | C | ABC | BC | A | D | AB | CD | *** | ns | ** |
| Hesperetin deoxyhexoside hexoside | A | A | A | A | B | B | B | B | ns | *** | ns |
| Hesperetin hexoside deoxyhexoside | A | A | A | A | B | B | B | B | ns | *** | ns |
| Kaempferol hexoside deoxyhexoside #1 | AB | B | AB | AB | A | AB | A | AB | * | * | ns |
| Kaempferol hexoside deoxyhexoside #2 | A | A | A | A | B | BC | B | C | * | *** | * |
| Kaempferol hexoside deoxyhexoside #3 | C | C | BC | C | A | C | AB | BC | *** | *** | ** |
| Isorhamnetin hexoside desoxyhexoside | A | A | A | A | B | B | AB | B | ns | *** | ns |
| Isorhamnetin methylhexose hexose | B | B | B | B | A | AB | A | A | ns | *** | ns |
| Apigenin deoxyhexose hexose | B | C | B | B | A | A | A | A | ** | *** | ** |
| Apigenin aglycone | A | A | A | A | B | B | B | C | *** | *** | *** |
| Tangeretin #1 | A | A | A | A | B | BC | B | C | * | *** | * |
| Tangeretin #2 | A | AB | A | A | B | B | B | C | *** | *** | *** |
| Tangeretin #3 | A | A | A | A | B | BC | B | C | * | *** | * |
| Scopolin | AB | B | AB | AB | A | AB | AB | C | *** | ns | *** |
| Scopoletin | A | ABC | AB | A | E | CDE | DE | BCD | ns | *** | ** |
| Caffeoyl quinic acid | AB | A | A | B | - | - | - | - | * | - | - |
| Nomilin deoxyhexoside | ABC | ABC | ABC | BC | CD | D | AB | A | * | ns | *** |
| Nomilin | A | B | B | A | - | - | - | - | * | - | - |
| Obacunone | A | A | A | A | B | B | B | B | ns | *** | ns |
| Limonin | A | A | A | A | B | B | B | B | ns | *** | ns |
| Lysophosphatidyl choline | A | A | A | B | B | B | AB | C | *** | *** | *** |
| Linolenic acid | AB | A | AB | A | B | AB | AB | B | ns | *** | ns |
| Linolenic acid hexoside hexoside | ABC | AB | A | A | CD | D | BC | AB | *** | *** | ** |

**Table S3**. Analysis of variance of semi-polar metabolite levels in Carrizo and Cleopatra plants in response to drought (WS), heat stress (HS) and the combination of drought and heat stress (WS+HS). Different letters denote statistical significance at p≤0.05. S: stress treatment; G: genotypes; SxG: interaction stress treatment x genotype. *p<0.05; **p<0.01; ***p<0.001; ns: no statistical differences.
