# Supplementary material for: Activation of Secondary Metabolism in Citrus Plants Is Associated to Sensitivity to Combined Drought and High Temperatures
Source: Front Plant Sci. 2017 Jan 9;7:1954. doi: 10.3389/fpls.2016.01954 (PMC5220112; doi:10.3389/fpls.2016.01954)

Figure S1. Normalized peak areas of detected secondary metabolites: quercetin, hesperetin and kaempferol derivatives. See Table S3 for more details on statistical analyses.

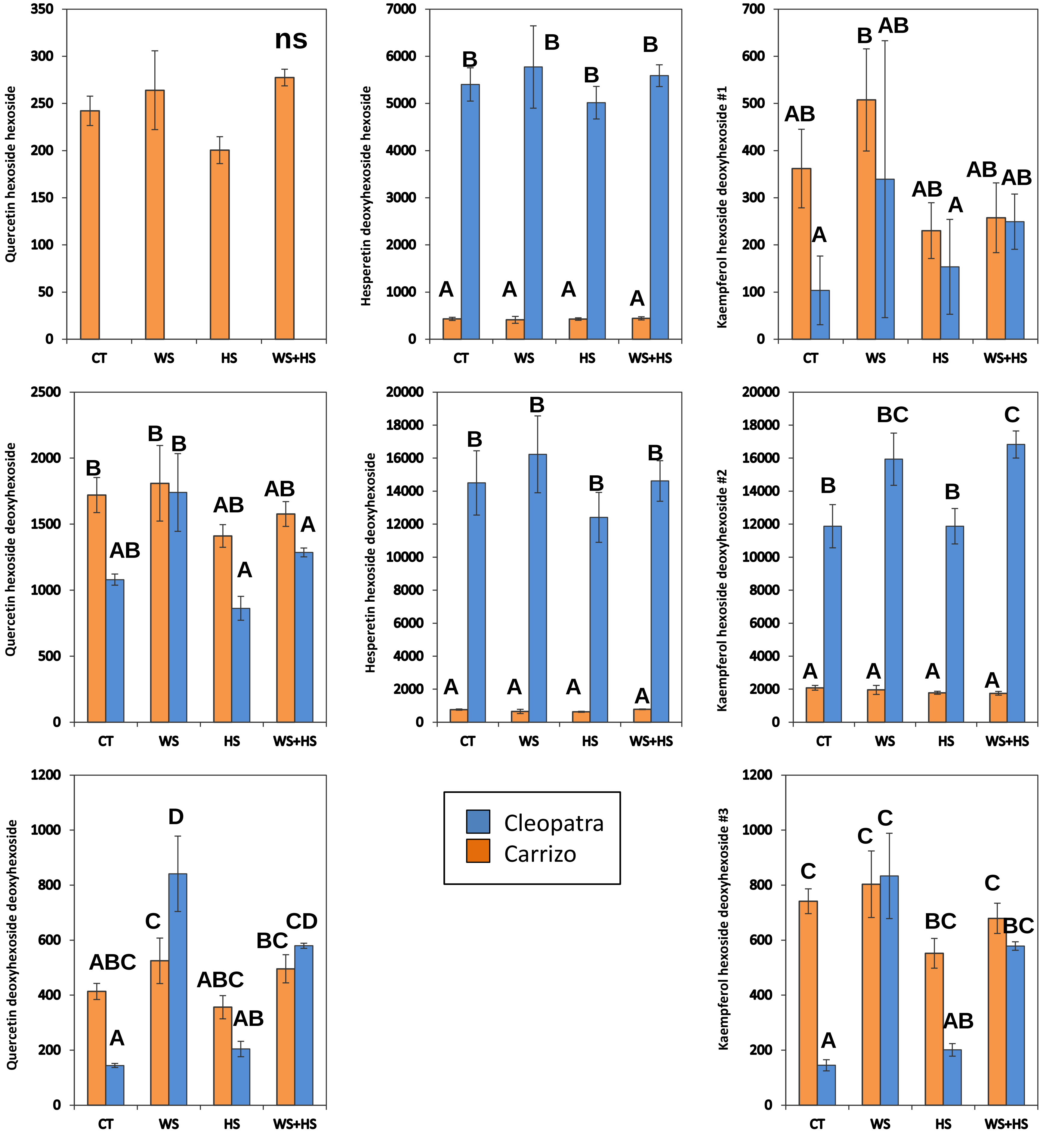

Figure S1. Normalized peak areas of detected secondary metabolites: isorhamnetin and apigenin derivatives and tangeretin isomers. See Table S3 for more details on statistical analyses.

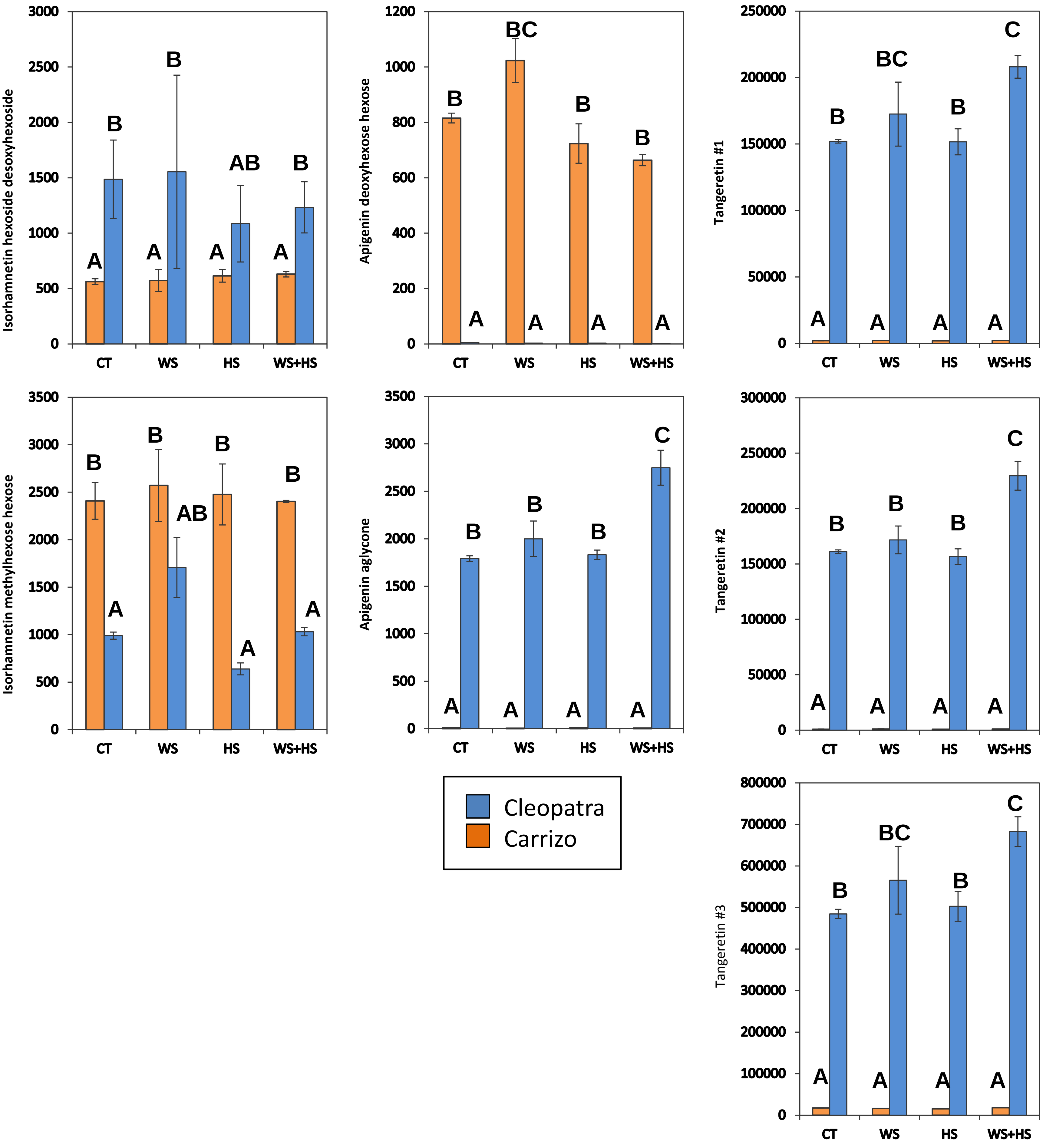

Figure S1. Normalized peak areas of detected secondary metabolites: citrus limonoids, fatty acids and derivatives. See Table S3 for more details on statistical analyses.

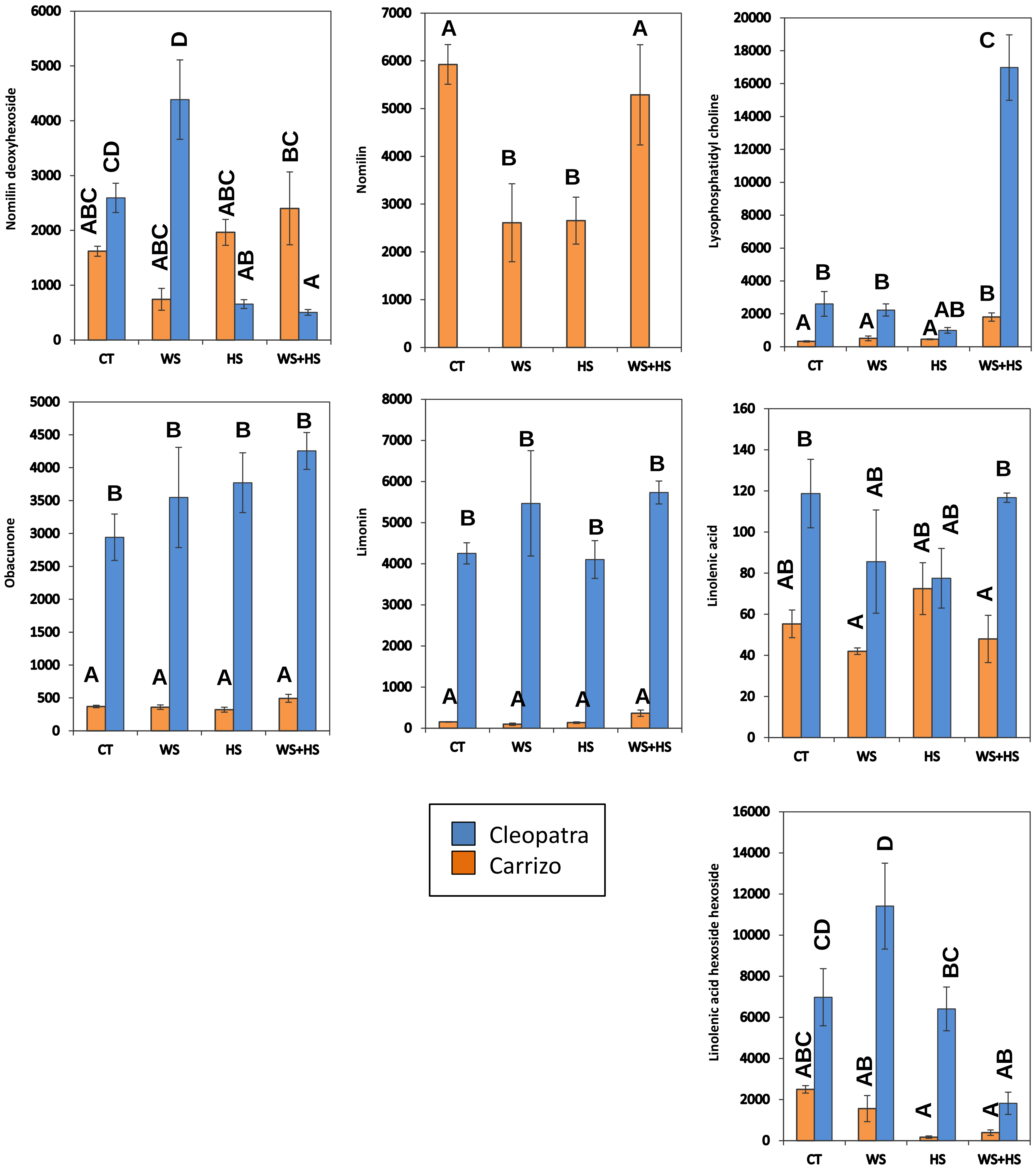

Supplement: Supplementary file 4 [file Image1.PDF]
