# Supplementary material for: Activation of Secondary Metabolism in Citrus Plants Is Associated to Sensitivity to Combined Drought and High Temperatures
Source: Front Plant Sci. 2017 Jan 9;7:1954. doi: 10.3389/fpls.2016.01954 (PMC5220112; doi:10.3389/fpls.2016.01954)

Figure S2. Normalized peak areas of tryptophan and tyrosine. Different letters denote statistical significance at p≤0.05. S: stress treatment; G: genotypes; SxG: interaction stress treatment x genotype. *p<0.05; **p<0.01; ***p<0.001; ns: no statistical differences.

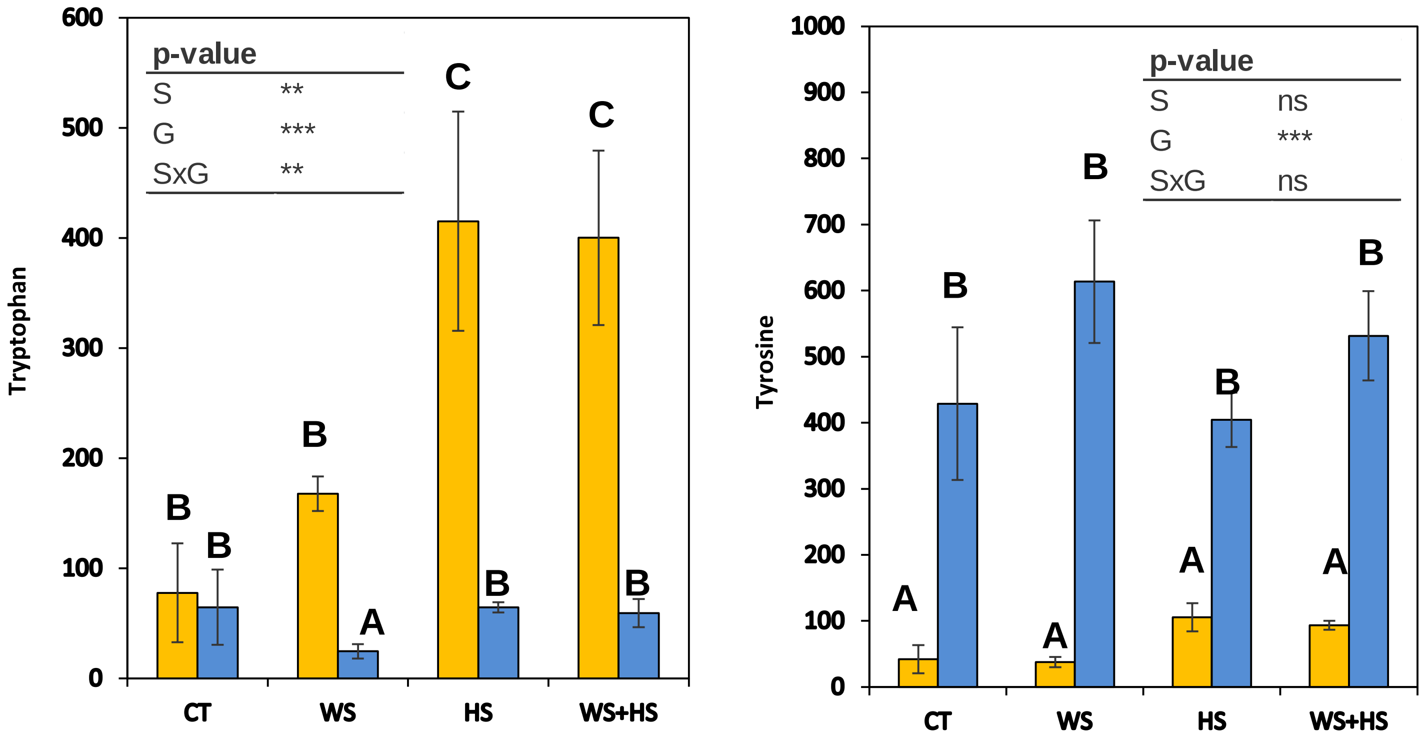

Supplement: Supplementary file 5 [file Image2.PDF]
